# Genome-wide identification of DNA methylation provides insights into the association of gene expression in rice exposed to pesticide atrazine

**DOI:** 10.1038/srep18985

**Published:** 2016-01-07

**Authors:** Yi Chen Lu, Sheng Jun Feng, Jing Jing Zhang, Fang Luo, Shuang Zhang, Hong Yang

**Affiliations:** 1 Jiangsu Key Laboratory of Pesticide Science, College of Sciences, Nanjing Agricultural University, Nanjing 210095, China; 2 Key Laboratory of Monitoring and Management of Crop Diseases and Pest Insects, Ministry of Agriculture, Nanjing Agricultural University, Nanjing, China; 3 Department of Biochemistry and Molecular Biology, College of Life Science, Nanjing Agricultural University, Nanjing 210095, China; 4 State key laboratory of food science and technology, Jiangnan University, Wuxi 214122, China

## Abstract

Atrazine (ATR) is a pesticide widely used for controlling weeds for crop production. Crop contamination with ATR negatively affects crop growth and development. This study presents the first genome-wide single-base-resolution maps of DNA methylation in ATR-exposed rice. Widespread differences were identified in CG and non-CG methylation marks between the ATR-exposed and ATR-free (control) rice. Most of DNA methyltransferases, histone methyltransferases and DNA demethylase were differentially regulated by ATR. We found more genes hypermethylated than those hypomethylated in the regions of upstream, genebody and downstream under ATR exposure. A stringent group of 674 genes (*p* < 0.05, two-fold change) with a strong preference of differential expression in ATR-exposed rice was identified. Some of the genes were identified in a subset of loss of function mutants defective in DNA methylation/demethylation. Provision of 5-azacytidine (AZA, inhibitor of DNA methylation) promoted the rice growth and reduced ATR content. By UPLC/Q-TOF-MS/MS, 8 degraded products and 9 conjugates of ATR in AZA-treated rice were characterized. Two of them has been newly identified in this study. Our data show that ATR-induced changes in DNA methylation marks are possibly involved in an epigenetic mechanism associated with activation of specific genes responsible for ATR degradation and detoxification.

Atrazine (1-Chloro-3-ethylamino-5-isopropylamino-2,4,6-triazine, ATR) is a pesticide widely used as a selective pre-emergence and post-emergence herbicide to improve crop (*e.g.* maize, sorghum, and rice) productivity. Due to its intensive use and moderate persistence in soils, ATR has become a contaminant in ground and surface water worldwide^1^. According to the reports, the concentration of ATR was detected at 21 μg L^−1^ in groundwater, 42 μg L^−1^ in surface waters, and 102 μg L^−1^ in river basins in agricultural areas^2^,^3^. ATR concentrations at 108 μg L^−1^ were also reported in the rivers of North America^4^. Some crops such as rice and corn were reported to absorb ATR readily from environments^5^,^6^,^7^. Accumulation of ATR caused abnormal plant growth and seriously physiological responses^7^,^8^,^9^. Overload of ATR in plants also induced homologous recombination of DNA^10^ and altered gene expression^11^,^12^,^13^. Plants have evolved various strategies to cope with the adverse impact of various xenobiotics; several mechanisms for catabolism and detoxification of pesticides have been proposed^14^. For instance, glutathione *S*-transferases (GSTs) and cytochrome P450 monooxygenases (P450s)-mediated catabolic processes have been implicated in the mechanism for degradation of toxicants^13^,^15^,^16^. However, the molecular and genetic regulatory mechanism for detoxification and degradation of pesticides in plants is largely unknown.

Recent genome-wide profiling of transcriptome has resulted in identification of many functional genes involved in pesticide accumulation, translocation, and degradation in plants^11^. Using high-throughput RNA sequencing, we have identified hundreds of functional genes related to degradation and detoxification of ATR in rice^12^. However, it is rarely known about the regulatory mechanism of gene expressions in response to ATR. Currently, the epigenetic mechanisms such as DNA methylation that control gene expression in environmental-stressed plants have been described^17^,^18^. DNA methylation may be an important mechanism involved in the regulation of plant response to organic contaminants.

DNA methylation is a biochemical process in which the methyl group is covalently attached to the 5-position of cytosine, yielding 5-methylcytosine (5-methylcytosine, 5mC). While the majority of DNA methylation events occur at CG, other nucleotide combinations such as CHG and CHH contexts are also the targets differentially methylated^19^. Addition of methyl groups (*e.g. S*-adenosine methionine) into cytosine is catalyzed by a group of enzymes known as DNA methyltransferases (DMT). To date, much of our understanding about DNA methylation mechanism in plants relies on the research on *Arabidopsis thaliana*. In rice, although there is a subset of methyltransferase families responsible for DNA methylation such as DNA methyltransferase 1 (MET1), domains rearranged methyltransferase (DRM) and chromomethyltransferases (CMTs)^20^, only a few of them have been genetically characterized. MET1 and its members act as maintenance methyltransferases responsible for introducing methyl groups specifically into CG sequences. The methyltransferase DRM2, guided by 21–24 nt small RNAs with ARGONAUTE4 (AGO4), is a homologue of the mammalian DNA methyltransferase 3 (DNMT3) family, catalyzing asymmetric CHH methylation through persistent *de novo* methylation^21^. CMTs are a group of plant specific DNA methyltransferases for maintenance of symmetrical CHG methylation^22^. In addition, there are several proteins or enzymes-coding genes such as *ROS1* (*REPRESSOR OF SILENCING*), *DME* (*DEMETER*), *DML2* (*DEMETER-LIKE*) and *DML3* responsible for the active demethylation of 5-methyl cytosine in plants^23^.

Rice is the major crop in many parts of the world and is commonly used as an experimental model to investigate plant response to organic toxicants^9^. Although there are reports indicating that ATR contamination of rice affects many aspects of physiological metabolisms^7^,^24^ and cereal production as well^25^, the epigenetic mechanism for DNA methylation regulated by organic toxicants in plants has not been described. To improve our knowledge on the toxic mechanisms, an understanding of global DNA methylation is required. The present study has identified the pattern of cytosine methylation in ATR-exposed rice using single-base-resolution bisulfite-sequencing (BS-Seq) technology. The altered DNA methylation patterns were presented in the genome of rice exposed to ATR. The transcriptome of ATR-exposed rice was profiled to figure out the link between DNA methylation and gene expression. To our knowledge, this is the first report on global DNA methylation modified by organic toxicants in plants.

## Results

### Bisulfite sequencing of rice genome exposed to atrazine

To get an insight into the methylation landscape, we performed a single-base DNA methylation bisulfite-sequencing of ATR and non-ATR (control or CK)-exposed rice samples. One week-old rice seedlings were exposed to 0 (control, CK) and 0.4 mg L^−1^ ATR for 0, 2, 4 and 6 d, and total DNA was extracted, respectively. For the control and ATR-treated samples, DNA extracted from each time point was pooled and sequenced. A total of 220 (CK) and 197 (ATR) million sequencing reads were generated with a high conversion rate of 99.6 and 99.7%, respectively (Supplementary Table S1). From the reads aligned, 71.3~115.3 M unique reads from both samples were filtered, yielding an average read depth of 17.2~27.7 X (per base for each DNA strand) (Supplementary Table S2). The overall unique reads of the two lines had an at least 80% coverage of all cytosines in the genome (Supplementary Fig. S1A). The effective coverage for chromosomes and intergenic regions was over >90% and >89% for the two samples, respectively (Supplementary Table S3), indicating that BS-Seq was sufficiently high-throughput. A locus (LOC_Os10g38470.1) was randomly selected for validation of BS-Seq quality. The DNA methylation marks of the locus were well confirmed by the specific PCR (Supplementary Fig. S2).

### Landscape of DNA methylation pattern in ATR-exposed rice

We identified 24.8 and 20.3 million mCs from all mapped reads of ATR-free and ATR-exposed rice, respectively. Identifying the whole levels of methylated and non-methylated cytosines showed that ATR exposure altered the landscape of DNA methylation (Supplementary Fig. S1B and C), with the cytosine methylation level (83.59%) over the control (81.68%) (Supplementary Fig. S1D). Under ATR exposure, the overall level of methylation in all sequence contexts including mC, mCG, mCHG and mCHH were increased as compared to the control. By analyzing the percentage of methylated cytosines across the two samples, we showed that approximate 51.8 ~ 52.9% mCs occurred at CG sites, 32.4~32.5% at CHG sites and 14.7~15.8% at CHH sites (Supplementary Fig. S1E), which is very similar to the recent report from rice^26^.

We further inspected the DNA methylation levels throughout the 12 chromosomes. Treatment with ATR induced a high level of CHH and CHG methylation, but in CG the methylation level was increased to a lesser extent (Supplementary Fig. S3A). The coordinating smoothed lines were displayed with mCG and mCHG at a 10 kilobase (kb) resolution, which has complementary distributional characteristics with mCHH sites. The local changes in DNA methylation levels were detected in the sequence context (Supplementary Fig. S4A). An example was known from chromosome 2 and 7, where the local methylation patterns at mCG, mCHG and mCHH between ATR-free and ATR-treated rice were clearly distinguished (Supplementary Fig. S3B-E). The methylcytosine levels were further examined in different regions of 3′-UTR, 5′-UTR, CDS, intron and mRNA. Methylation at CHG and CHH sites with ATR was higher than that of the ATR-free (Supplementary Fig. S4A, right). Moreover, DNA methylation between CG and non-CG sites was profiled using a 200 bp sliding window. ATR exposure altered the local DNA methylation levels in the specific regions (Supplementary Fig. S4B).

### ATR exposure alters global DNA methylation patterns of genes from rice

**Figure 1:**
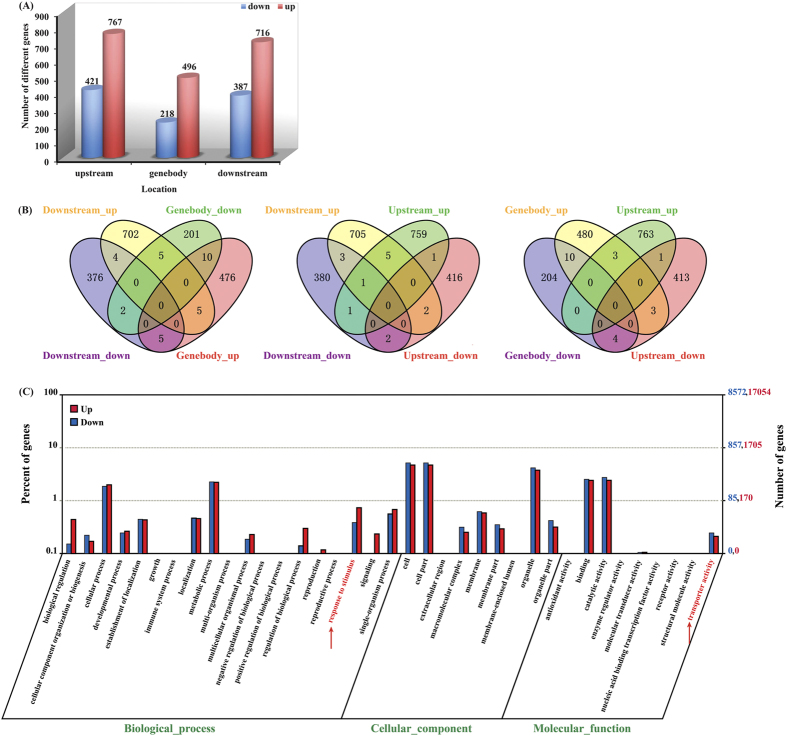
Differentially methylated genes (DMGs) and gene ontology (GO) enrichment analysis. (**A**) DMGs in different genomic regions (upstream, genebody and downstream). Differentially methylation patterns were characterized from the difference of methylcytosine levels. (**B**) Venn diagrams display the overlap between the up-regulated or down-regulated methylation genes in different genomic regions (upstream, genebody and downstream) under ATR exposure. (**C**) Go clustering analysis of DMGs. Annotations are grouped by molecular function or biological process based on the rice GO annotation information.

Search for genomic regions associated with CG, CHG and CHH methylation marks resulted in identification of a total of 2831 ATR-regulated differentially-methylated regions throughout the 12 chromosomes (Supplementary Table S4), from which 3007 non-redundant ATR-regulated differentially-methylated genes (DMGs) were identified (Fig. 1A). Of these, 1979 genes were hypermethylated and 1026 were hypomethylated, indicating that more genes tended to be methylated under ATR exposure. Further examination of the methylated regions of upstream, genebody and downstream revealed that the hypermethylation or hypomethylation occurred specifically for the vast majority of the genes, and very few genes were found to be redundant between hypermethylation and hypomethylation (Fig. 1B). This is in agreement with the previous studies that the regulatory region of genes is the major target of methylation/demethylation^19^,^21^. Compared to upstream and downstream regions, genebody appeared to be a region of more flexible methylation targets.

We further employed the Web Gene Ontology Annotation Plotting (WEGO) to functionally categorize the DMGs. A strong enrichment in different biological processes was observed (Fig. 1C). According to the specific categories, these genes can be classified into three major groups including biological process, cellular component and molecular function. In biological process, a high cluster pointed to the cellular and metabolic processes and response to stimulus. Notably, some of the DMGs were enriched in functions associated with transport activity, suggesting possible involvement in ATR uptake and translocation in rice plants.

### Many functionally methylated genes are regulated by ATR

**Figure 2:**
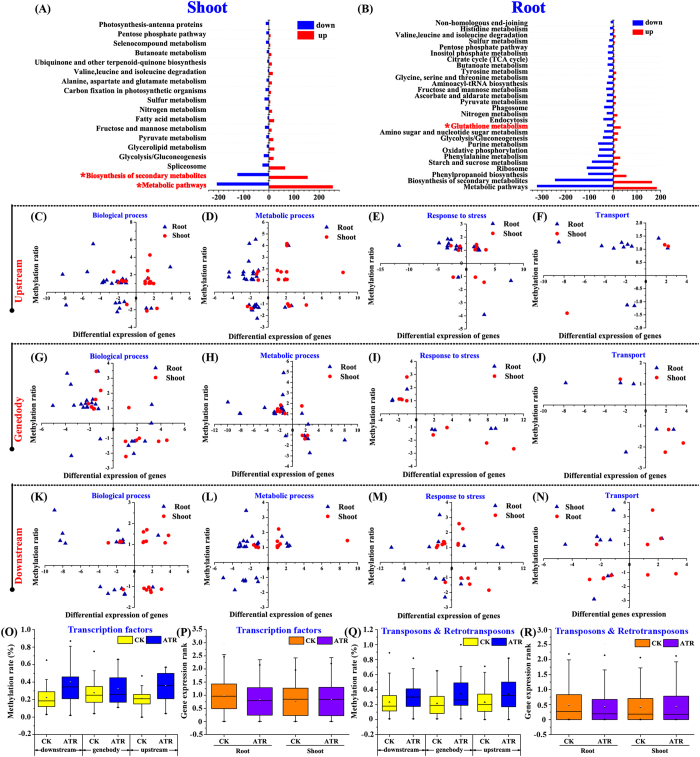
Transcriptome analysis of ATR-exposed rice. (**A,B**) Enrichment of significant pathways (*p*-value < 0.05, Q value < 0.05) in KEGG in each pair of rice libraries (shoot + ATR vs shoot-ATR, root + ATR vs root-ATR). (**C~N**) The relations between DNA methylation and mRNAs at various genomic regions. Differential expression of genes (|Log_2_TPM_treatment_/TPM_CK_| ≥1, *y* axis) as a function of DMGs (|Log_2_μ_treatment_/μ_CK_| ≥1, *x* axis) in hierarchical clustering (biological process, metabolic process, response to stress and transport). DNA methylation rates of transcription factor-coding genes (**O, P**) and transposon/retrotransposon (**Q, R**). Boxes in **O** to **R** represent the quartiles and whiskers marking the minimum and maximum values.

To investigate whether ATR-regulated DNA methylation marks was associated with gene expression, we profiled the transcriptome prepared from the same and samples used for BS-Seq. Transcriptome in roots and shoots was separately analyzed because the homogeneous genomic DNA methylation occurs in rice seedlings^27^. To ensure the genes that were differentially expressed, ten genes were randomly selected for qRT-PCR validation. All genes were well confirmed in expression (Supplementary Fig. S5). The two-fold change (or more) threshold was used to screen genes differentially expressed and methylated (*p* < 0.05) under ATR exposure. In total, 674 genes passed through the criteria. There were 182/132/141 genes up-regulated and 102/51/81 genes down-regulated in methylation in the upstream, genebody and downstream regions, respectively (Supplementary Table S5; Supplementary Fig. S6). All genes were identified by Blastx searching against Kyoto Encyclopedia of Genes and Genomes (KEGG) databases (http://www.genome.jp/kegg/pathway.html). The major pathways that the genes were grouped in concern the biosynthesis of secondary metabolites and metabolic pathway (Fig. 2A,B). Some genes were also found involving the pathway of glutathione metabolism, which is implicated in the mechanism for ATR detoxification^13^.

The relationship between DNA methylation status and relevant gene expression was presented by plotting two-dimensional gene distribution. Some genes were chosen from biological process, metabolic process, transport, and stress response. Genes whose upstream regions were hypermethylated tended to be down-regulated by ATR (Fig. 2C–F). It is noted for those whose genebody with hypermethylation or hypomethylation was negatively associated with gene expression (Fig. 2G–J). This observation is very similar to the previous studies^27^. However, no clear association was observed between the methylation at the terminal regions and gene transcription (Fig. 2K–N). We further identified the association between DNA methylation and expression for all transcription factor (TF)-coding genes and transposon/retrotransposon. Genes for TF usually had higher methylation at their upstream and downstream under ATR exposure, which led to a general decrease in the gene expression in roots (Fig. 2O,P). This case was true for transposon and retrotransposon in ATR-exposed rice (Fig. 2Q,R).

**Figure 3:**
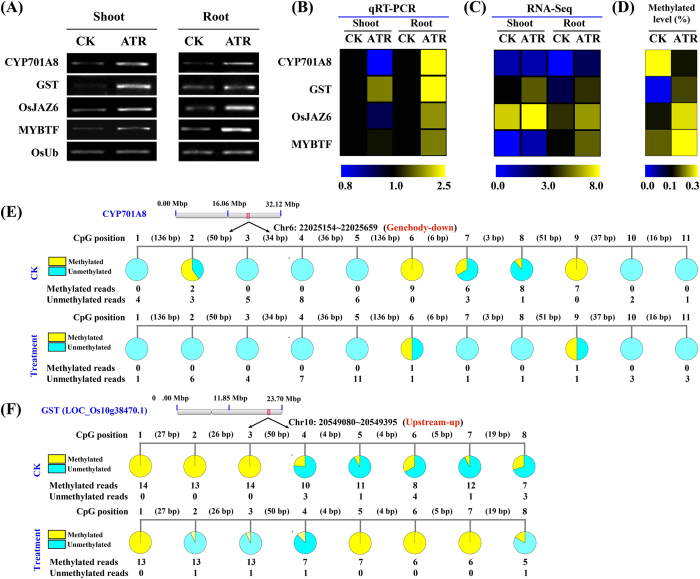
Representative ATR-responsive genes with variations in DNA methylation and gene expression. Validation of gene expresison by semi-quantitative RT-PCR (**A**) and by qRT-PCR (**B**). (**C**) RNA-Seq (≥2-fold change, *p* < 0.05). Data were presented as the ratio of Log_2_ [values (μ_atrazine-treated_ /μ_control_)]. (**D**) methylated levels determined by BS-Seq (≥2-fold change). Four loci are LOC_Os06g37300 (*CYP701A8*), LOC_Os10g38470 (glutathione *S*-transferase gene, GST), LOC_Os03g28940 (*OsJAZ6*) and LOC_Os07g48870 (MYB transcription factor). Semi-quantitative RT-PCR and qRT-PCR validation of gene expression in rice exposed to ATR at 0 and 0.4 mg L^−1^ for 2, 4 and 6 d. The treated samples were pooled and analyzed. Rice ubiquitin was used as an internal reference gene for normalization. Experiments were repeated in triplicate (*p* < 0.05, ANOVA).

To further demonstrate the connection between methylation and gene expression, we profiled four randomly selected genes including (a) LOC_Os06g37300 coding for a putative cytochrome P450 monooxygenase or *CYP701A8*, (b) LOC_Os10g38470 (encoding putative GST) possibly involved in organic toxicant degradation and detoxification^13^,^28^, (c) LOC_Os03g28940.1 (*OsJAZ6*) involved in biotic stress response^29^, and (d) LOC_Os07g48870.1 encoding a putative MYB transcription factor. Semi-quantitative RT-PCR and qRT-PCR analyses revealed that the four genes were induced by ATR (Fig. 3A–C), and were also differentially methylated under ATR exposure (Fig. 3D). We further profiled the DNA methylation marks in their specific regions (Fig. 3E,F; Supplementary Fig. S7). The *CYP701A8* genebody region was found to be hypomethylated. The lower DNA methylation level was associated with increased expression of the gene under ATR exposure. In contrast, the upstream regions of *OsJAZ6*, GST and MYBTF genes were hypermethylated under ATR stress. Interestingly, these genes were also induced under ATR exposure. Besides, 14 genes encoding essential metabolic enzymes that involved in ATR-degradation (*e.g.* cytochrome P450, dehydrogenase, hydrolase and GST) showed upregulated expressions and differential methylation levels (Supplementary Fig. S8).

### ATR alters expression of genes associated with DNA methylation

We identified genes responsible for DNA methylation/demethylation from rice RNA-Seq datasets and analyzed their expression abundance with ATR exposure. Rice contains ten DNA methyltransferase family members and can be functionally classified into several subfamilies (Supplementary Fig. S9)^30^,^31^. In this study, six genes encoding DNA methyltransferases were identified and showed differential regulation by ATR (Supplementary Fig. S9). *DMT707* (LOC_Os07g08500, *OsMET1-2*) belongs to the MET (DNA methyltransferase) family, playing an important role in maintaining DNA methylation marks^32^. Expression of *DMT707* in rice was repressed under ATR exposure. *DMT703* (LOC_Os05g13790) and *DMT704* (LOC_Os10g01570) encode chromomethylase (CMT) family methyltransferases responsible for CHG and are only found in plants^21^,^22^. While expression of *DMT703* was enhanced, *DMT704* was strongly repressed in expression with ATR. The last part of DNA methyltransferase subfamily, including *DMT705* (LOC_Os01g42630), *DMT706* (LOC_Os03g02010, *OsDRM2*) and *DMT710* (LOC_Os05g04330), is the domains rearranged methyltransferase (DRM) responsible for *de novo* DNA CHH-specific methylation. *DMT705* and *DMT706* had a similar expression pattern with weak enhancement in shoots and repression in roots, while *DMT710* had an opposite expression pattern. These results indicate that a variety of DNA methyltransferase could be differentially regulated by ATR.

Recent studies show interplay between DNA methylation and histone modifications^33^. For example, the KRYPTONITE H3K9 histone methyltransferase is required for CHG and CHH DNA methylation in *Arabidopsis*^34^. The global transcriptome and mutant analysis has identified many genes involved in histone-related methylation marks, chromatin remodeling (such as DDM1 required for the maintenance of CG and non-CG methylation), RNA-directed DNA methylation (RdDM), and polycomb repressive complex 2 proteins-coding genes in rice and other plant species^30^. *OsDDM1a*, *OsDDM1b* and *OsCHR4* are a subset of genes responsible for chromatin remodeling^30^. Under ATR exposure, *OsDDM1a* and *OsDDM1b* were induced in shoots, but repressed in roots. Expression of *OsCHR4* was also repressed in ATR-exposed rice. *SDG714* is encoding a histone H3K9 methyltransferase^35^. Expression of *SDG714* was strongly induced by ATR in shoots, but it was undetectable in roots. *SDG725* is encoding a histone H3K36 methyltransferase^36^,^37^. Expression of *SDG725* in rice plants was repressed by ATR. Notably, two genes *JMJ703* and *JMJ706* encoding H3K4 demethylase were differentially regulated by ATR, suggesting the involvement in the ATR signaling of DNA methylation.

We identified several RNA-dependent RNA polymerase (RDR) protein genes generating the 24 nt small RNAs (sRNAs). RDR1/2/4/6 are required for RdDM, a plant specific *de novo* DNA methylation pathway for transcriptional silencing of transposable elements and other DNA repeats to maintain genome stability^38^. In similar, these genes were differentially regulated by ATR in rice. Polycomb repressive complex 2 proteins-coding genes are encoding H3K27 methyltransferases responsible for plant elongation, floral organ development^39^. Interestingly, most of the genes including *OsiEZ1*, *OsCLF*, *OsFIE2* and *OsEMF2a* in shoots were induced, but repressed in roots by ATR exposure.

### ATR alters DNA methylation marks of transposable and retrotransposable elements

Since DNA hypermethylation occurring to the regions of transposable elements could be interpreted as the enhancement of genome stability, the DNA methylation regions linked to the transposable and retrotransposable elements were identified. There were 47 transposons and 127 retrotransposons differentially methylated under ATR stress (Supplementary Fig. S10A). Under the same condition, 238 transposons (83 in shoot; 155 in root) and 502 retrotransposons (197 in shoot; 305 in root) were differentially regulated (Supplementary Fig. S10B). While the transposons/retrotransposons had a higher methylation level, they usually showed a generally reduced transcription levels in tissues, particularly in roots under ATR exposure (Fig. 2Q,R).

We further identified the transposons and retrotransposons that were both differentially methylated (≥2-fold, *p* < 0.05) and differentially expressed (Log_2_|fold change| ≥1, *p* < 0.05). There were 3 transposons (two expressed only in root and one in both root and shoot) and 5 retrotransposons (all expressed in root) that met the criteria (Supplementary Fig. S10C). Their transcript level was presented in Supplementary Fig. S10D.

There were 1012 genes located nearby the transposons/retrotransposons. Of these, 30 genes were differentially expressed (Log_2_|fold change|≥1, *p* < 0.05) only in root, 145 genes only in shoot, and 178 genes in shoot and root (Supplementary Fig. S10E). GO analysis showed that the top enriched gene functions included binding, catalytic activity, cellular process and metabolic process (Supplementary Fig. S10F). Finally, combinational analyses with DNA methylation revealed 16 transposons- and 10 retrotransposons-nearby genes that were differentially expressed and methylated (≥2-fold, *p* < 0.05) (Supplementary Fig. S10G).

### Genetic validation of ATR-responsive DNA methylation associated with gene expression

**Figure 4:**
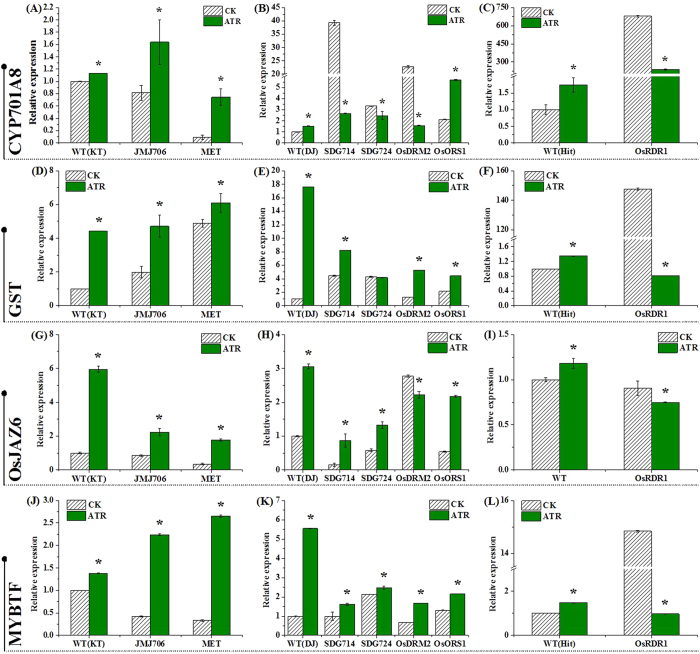
qRT-PCR analysis of transcripts of four genes in ATR-exposed rice mutants. Seven day-old seedlings were treated with 0.4 mg L^−1^ atrazine for 4 d, and total RNA was extracted for qRT-PCR analysis. The tested genes included LOC_Os06g37300.1, *CYP701A8*; LOC_Os10g38470.1 encoding glutathione *S*-transferase, GST; LOC_Os03g28940.1, *OsJAZ6*; and LOC_Os07g48870.1 encoding MYB transcription factor, MYBTF. The background of the mutants and their wide-type was presented as Dong Jing (DJ), KT and Hitomebore (Hit), respectively. Error bars indicate (±) SD with three replicates. Means followed by asterisks are significantly different between the mutants and wild-type under ATR stress (*p* < 0.05, ANOVA).

We used a subset of mutants defective in activities of DNA methylation/demethylation, histone modification and small RNAs to assess transcript levels of selected genes described in Fig. 3. The MET1 gene (encoding CG methyltransferase) is required to maintain CG methylation; mutation at the locus results in elimination of CG methylation^33^. Compared to its wide type, *met1* usually had a higher transcript abundance of the tested genes under ATR stress (Fig. 4A,D,G and J). The rice *JMJ706* encodes a heterochromatin-associated H3K9 demethylase; mutation of *JMJ706* leads to increased di- and trimethylations of H3K9 and affects the spikelet development^40^. Similar to *met1*, the transcript level of the four genes also increased under ATR exposure. *SDG714* (*SUVH6*) and *SDG724* encode H3K9 and H3K36 methyltransferases for histone methylation, respectively^35^,^37^. Mutation of *SDG714* (KRYPTONITE *SUVH6*) and *SDG724* led to repression of *CYP701A8* under ATR exposure (Fig. 4B), suggesting that both genes are possibly regulated by DNA methylation through the histone methylation mechanism. However, the mutation of *SDG714* and *SDG724* seem not to affect the expression of three other genes (Fig. 4E,H and K).

The domains rearranged methyltransferases are *de novo* methyltransferases required for CHH methylation to target sites in a fashion of being guided by small RNAs through the process of RdDM in plants^41^. Compared to wide-type, mutation of *DRM2* reduced transcripts of *CYP701A8* and *OsJAZ6* under ATR stress (Fig. 4B,H), but did not affect transcription of LOC_Os10g38470.1 and LOC_Os07g48870.1 (Fig. 4E,K). We also examined *ros1* and did not find any effect on expression of the genes (Fig. 4B,E,H and K). *OsRDR1* was tested because recent evidence showed that *OsRDR1* is involved in regulation of genome-wide changes in gene expression, regional variation in small RNAs and alteration in DNA methylation in rice^38^. Interestingly, all genes identified here were found to be repressed by ATR exposure (Fig. 4C,F,I and L). These results suggest that only some, but not all of the epigenetic-modified genes (such as genes coding for methyltransferases) were involved in the regulation of the gene expression.

### Effect of a DNA methylation inhibitor on plant growth, ATR accumulation and formation of ATR metabolites in rice

**Figure 5:**
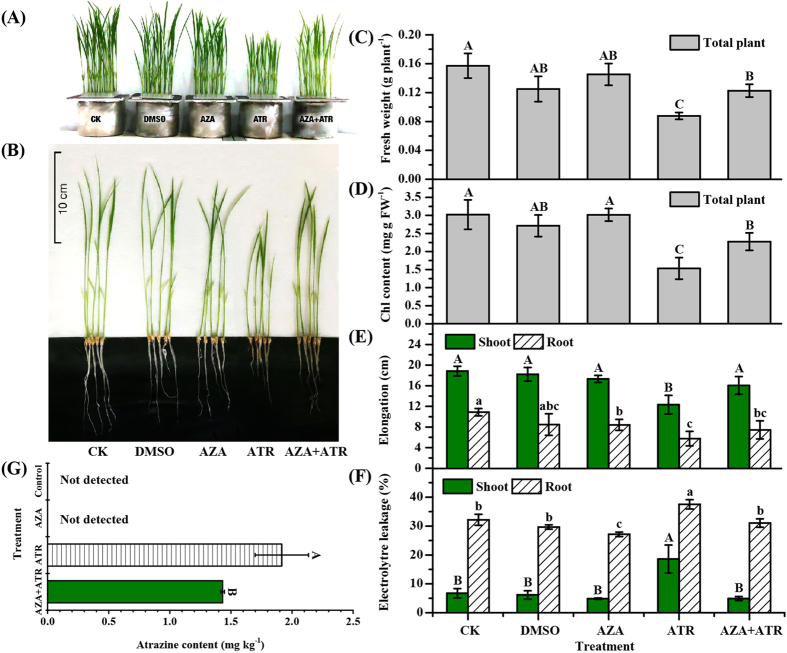
Effects of DNA methyltransferase inhibitors 5-azacytidine on growth and ATR accumulation of rice. Seven day-old seedlings were pretreated with 20 μM 5-azacitidine (AZA) for 16 h and then exposed to 0.4 mg L^−1^ atrazine for 4 d. (**A,B**) Phenotypes of growth; (**C**) biomass (fresh weight); (**D**) chlorophyll content; (**E**) shoot and root elongation; (**F**) membrane permeability; and (**G**) ATR content in plants. Values are the means ± SD with three replicates. Means followed by different capital letters (shoot), lower case letters (root) and asterisks are significantly different within the treatments (*p* < 0.05, ANOVA).

To investigate whether DNA methylation affected functional consequences such as growth, ATR accumulation in rice, a commonly-used DNA methylation inhibitor 5-azacytidine (AZA) was employed^42^. One week-old seedlings were subjected to a combinational treatment with 0.4 mg L^−1^ and 20 μM AZA. AZA addition was able to promote the growth and root/shoot elongation (Fig. 5A–C and E), increase chlorophyll contents (Fig. 5D), and lower membrane permeability (Fig. 5F). Moreover, provision of AZA could significantly reduce ATR content in rice as compared to the control (ATR treatment alone) (Fig. 5G).

**Figure 6:**
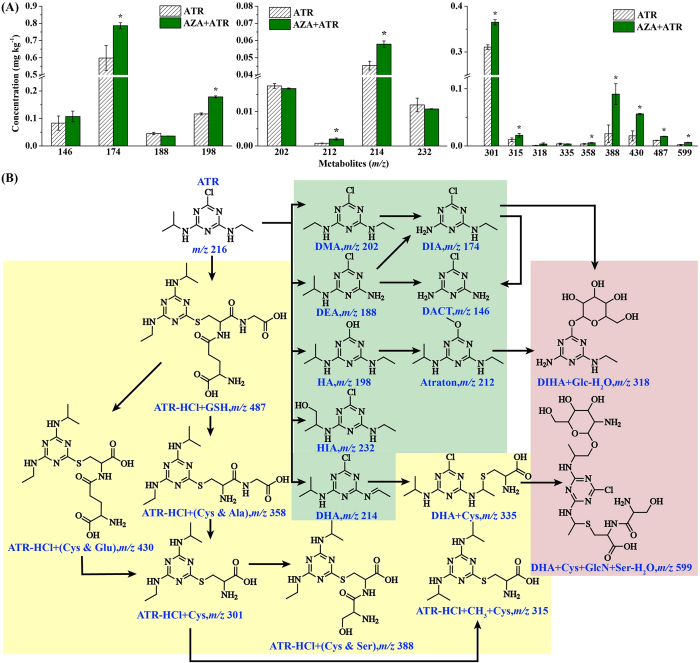
The content of ATR-metabolites detected by UPLC/Q-TOF-MS/MS and a proposed pathway of ATR metabolites in rice exposed to ATR and/or AZA. Seven day-old rice seedlings were exposed to ATR (0.4 mg L^−1^) and/or 5-azacytidine (AZA) for 4 d. After that, the metabolites were extracted and analyzed. Values are the means ± SD with three replicates. Asterisks are significantly different between ATR and AZA + ATR treatments (*p* < 0.05, ANOVA).

We further characterized chemical structures of degradation products and conjugates of ATR in AZA-treated rice using UPLC/Q-TOF-MS/MS. The accurate mass data ( < 5 parts per million errors) by high resolution MS were applied to confirming elemental formula. Isotope (M + 2) was monitored for identifying the chlorine-containing ATR transformation products. A total of 8 degraded products (including HIA, DHA, Atraton, DMA, HA, DEA, DIA, and DACT) and 9 conjugates (DHA + Cys & Ser + GlcN-H_2_O, ATR-HCl + GSH, ATR-HCl + (Cys & Glu), ATR-HCl + (Cys & Ser), ATR-HCl + (Cys & Gly), DHA + Cys, DIHA + Glc-H_2_O, ATR-HCl + CH_3_ + Cys and ATR-HCl + Cys) in ATR/AZA-exposed rice have been successfully characterized. The mass spectrometric data were summarized in Supplementary Table S6. According to the extracted ion chromatograms by full-scan acquisition of treated rice samples, signals of ATR and its metabolites were detected in ATR- and AZA + ATR-treated rice samples, but not in the control (ATR-free and AZA-free) (Supplementary Fig. S11). Seven ATR-glutathione metabolites and two glucosylated-ATR conjugates were identified. In comparison with the control (ATR-treatment alone), more ATR-glutathione metabolites and glucosylated-ATR conjugates were detected (Fig. 6). An obvious finding was that more ATR-derivatives were detected in AZA + ATR-treated rice than those in ATR-treated rice (Fig. 6A), indicating that AZA application was able to accelerate the metabolism of ATR in rice. Of the 8 degraded products and 9 conjugates, Atraton is only reported in animals, and DHA + Cys & Ser + GlcN-H_2_O and ATR-HCl + (Cys & Ser) are reported here for the first time. The detailed description about the chemical analyses was included in Supplementary Note. These data indicate that modification of DNA methylation can finally affect ATR metabolic products.

### Identification of loci generating microRNAs differentially methylated under ATR stress

MicroRNAs (miRNAs) are a class of non-coding small RNAs with about 21 nt long and transcribed by RNA polymerase II^43^. To investigate whether rice miRNA loci were methylated under ATR exposure, we retrieved all rice miRNA precursors from the publicly available database (http://www.mirbase.org/index.shtml) and mapped the precursor sequences to our bisulfite-sequenced datasets. With two-fold filter criterion, 15 miRNA-generating loci (representative of 10 miRNA families) were identified (Table 1). All these miRNA loci were hypermethylated under ATR exposure (≥2-fold change). As most of the miRNA targets are not identified in rice, we predicted the targets for the miRNAs. miR394 were predicted to target an F-box protein that has been identified in *Arabidopsis thaliana*^44^ and a transposon protein. Importantly, several glycosyltransferase genes were predicted for OsmiR1437b, OsmiR1862d and OsmiR1862e, respectively. In addition, a cytochrome P450 gene was predicted as a potential target of OsmiR812q. Furthermore, a putative ABC transporter was predicted to be a target of OsmiR5817. These miRNA targeted genes may be involved in ATR transformation and degradation in rice plants.

## Discussion

Growing evidence shows that environmental contaminants such as pesticides and other organic toxicants are able to modify DNA methylation marks in animals and human cells^45^,^46^. For example, Benzo[a]pyrene (BaP), a carcinogen and reproductive toxicant, can impair egg production of zebrafish and larvae by reducing global DNA methylation^47^. However, no report is available in plants about DNA methylation mediated by organic toxicants. We recent performed a genome-wide analysis of transcriptome in rice exposed to ATR and demonstrated that a large number of genes regarding Phase I to III detoxification and degradation pathway under ATR exposure, including genes encoding glycosyltransferases, cytochrome P450 monooxygenase, glutathione *S*-transferases, glycosyl hydrolase, laccase, etc^12^,^13^. Importantly, a good set of methylase- and methyltransferase-coding genes in response to ATR were identified. These studies promoted us to further characterize the global specific DNA methylation pattern and its correlation with the genes for ATR detoxification and degradation in rice. The present study showed that there are different mC marks between the ATR-free and ATR-exposed rice.

DNA methylation and demethylation are associated with gene expression^48^. A combinational analysis of global methylation and transcriptional changes was conducted. Alignment of significant genes ( > 2-fold changes in methylation and expression) revealed that 674 genes can be differentially methylated under ATR exposure. Identification of the differentially methylated genes showed that there were more DMGs hypermethylated than those hypomethylated in the upstream, genebody and downstream, indicating that the specific DNA methylation was modified by ATR exposure. Many of the genes were found to be involved in metabolic process, biosynthesis of secondary metabolites, and several other pathways such as stress response and transport, suggesting that ATR-mediated DNA methylation is closely associated with specific genes responsible for ATR metabolism or degradation. We further specified DNA methylation and gene expression by profiling the methylation marks of upstream, genebody and downstream regions of four loci. For example, the genebody of *CYP701A8* was hypomethylated. Such a DNA methylation pattern could be the cause of the enhanced expression of *CYP701A8 *under ATR exposure. This observation was genetically supported by a subset of mutants defective in specific DNA methylation activities. The *CYP701A8* expression in most of the mutants was depressed (except *jmj706 *and *met1*). Furthermore, *CYP701A8* in *ros1* was substantially induced by ATR, suggesting that both methylation and demethylation were possibly involved. Plant P450s constitute one of the largest protein family involved in plant acclimation to biotic and abiotic stresses^49^. In higher plants, the pesticide metabolism comprises three major active phases including conversion (Phase I), conjugation (Phase II), and compartmentalization (Phase III); P450s is thought of playing a critical role in the phase I metabolism^15^. We recent performed a global identification of P450 genes and assessed their activity that acted on atrazine. Importantly, an array of degradation products has been chemically characterized using by ultra performance liquid chromatography mass spectrometry (UPLC/MS). Furthermore, two specific inhibitors of piperonylbutoxide (PBO) and malathion (MAL) were used to assess the correlation between the P450s activity and rice responses including accumulation of atrazine in tissues, shoot and root growth and detoxification^16^. These results indicate that some of P450s genes were involved in degradation of ATR in rice. In this study, a variety of ATR-degraded products in rice was identified, indicating that DNA methylation was involved in the metabolism or degradation of ATR in rice.

To investigate whether DNA methylation affects physiological responses, a commonly used DNA methylation inhibitor AZA was tested in this study^42^. Treatment with AZA improved the plant growth with regard to biomass, chlorophyll contents and tissue elongation (shoot and root) under ATR exposure (Fig. 5A–E). In addition, the eletrolytre leakage representing the damage of plasma membrane in rice was attenuated by AZA in the presence of ATR (Fig. 5F). Interestingly, seedlings with AZA accumulated less ATR than those treated with ATR alone (Fig. 5G). These results indicate that improvement of plant growth and less ATR accumulation in rice were attributed to DNA methylation or demethylation.

Because rice plants treated with AZA had a low level of ATR, this could be the result of metabolism or degradation of ATR. By using UPLC/Q-TOF-MS/MS, an array of degraded products and conjugates of ATR was characterized. There were eight ATR degraded products (HIA, DMA, Atraton, DHA, HA, DEA, DIA and DACT) belonging to phase I reaction, by which the toxicants can be activated through oxidoreduction and hydrolysis reaction^15^. Hydroxyisopropylatrazine (HIA) was ionized to yield characteristic product ions of *m/z* 214 (loss of H_2_O), but formed different ions of *m/z* 174 through loss of the N-hydroxyethyl moiety. The atrazine-hydroxylated product was proved to be non-phytotoxic to plants, as *in vivo* studies in vertebrates and human cells indicate that it is a detoxified product, which was likely catalyzed by cytochrome P450 enzymes^50^. Notably, the product Atraton formed by loss of the chlorine group and addition of the methoxy group is reported here for the first time in plants. It is generally considered that phase I is the most important step, by which an herbicide can be activated through oxidoreduction and hydrolysis reaction or catalyzed by cytochrome P450^16^. Here, four ATR-inducible genes encoding cytochrome P450 were found to be differentially methylated (Supplementary Fig. S8). Of the phase I reaction products, DHA and DIA were brought in phase II reaction, by which the products underwent the conjugation with polar donor molecules, such as glutathione, sugars, amino acids or others^14^. While DHA was conjugated with cytosine, it took several steps (*e.g.* chlorine was replaced by hydroxy) for DIA to be transformed into DIHA + Glc + H_2_O. In the phase II reaction group, we identified six ATR compounds that were generated by direct conjugation of ATR with polar donor molecules. One complex substance (ATR + HCl + GSH) was formed by interaction with glutathione (GSH), and five was involved in cytosine (Cys) or other types of amino acid. Obviously, the five conjugates were derived from ATR + HCl + GSH. γGlu-Cys-Gly (glutathione; GSH) is a thiol redox tripeptide and widely exists in plants and animals^51^. The conjugates with GSH can be hydrolyzed to Cys-conjugates by action of *γ*-glutamyl transpeptidases and Cys-Gly dipeptidases in succession. Two conjugates of ATR with GSH have been identified in *Medicago sativa*^52^. One is ATR-γGlu-Cys-Gly and the other is ATR-γGlu-Cys-βAla (homoglutathione; hGSH). Both have similar functions such as transport and storage of sulfur, scavenging of ROS, and detoxification of toxicants^53^. The conjugate of ATR with GSH was detected in the form of ATZ-HCl + GSH, suggesting a process of ATR detoxification. As GSTs catalyze the transfer of glutathione to a co-substrate with a reactive electrophilic center to form *S*-glutathionylated product, the GST-conjugates are usually processed by Phase III pathway (conjugates transfer to vacuoles or other subcellular fractions), thus making the plant detoxified^14^,^28^. Our recent studies showed that both GST transcription and enzyme activity were induced by pesticides in rice and other plants^7^. In this study, one of the putative GSTs could be hypermethylated under ATR stress. It should be interesting to identify further the genetic relation between the gene expression and DNA methylation.

The functional roles of miRNAs mainly concern the regulation of messenger RNA expression, stability or translation^46^. Recently, several studies have shown that some insecticides such as dichlorvos, fipronil and triazophos, induced expression of miRNAs in animals^54^,^55^. In plants, a group of miRNAs have been identified in response to environmental contaminants such as heavy metals^56^. However, whether miRNAs can be induced by organic toxicants and whether the toxicants-induced miRNAs can be meditated by DNA methylation in plants remain unknown. This study identified 15 miRNA loci that were differentially methylated under ATR exposure. Based on the predicted targets, most of the miRNAs are involved in stress response and detoxification. It is noted for several miRNAs such as OsmiR812q, whose target was predicted as a cytochrome P450 gene. Furthermore, several glycosyltransferase genes were predicted for OsmiR1437b, OsmiR1862d and OsmiR1862e. These miRNAs are potentially involved in ATR detoxification and degradation in rice.

In conclusion, this study identified a group of ATR-induced genes that can be differentially methylated in rice. This suggests that specific DNA methylation/demethylation during ATR exposure are directly or indirectly associated with the activation of functional genes responsible for ATR degradation or detoxification. Evidence has been provided that expressions of several genes such as *CYP701A8* and LOC_Os10g38470.1 under ATR stress were regulated by DNA methylation. We also showed evidence that genome-wide DNA demethylation due to AZA inhibitor application led to changes in degradation products of ATR in plants. These results indicate that DNA methylation affects not only the expression of genes for ATR degradation/detoxification but the physiological consequences as well.

## Materials and Methods

### Plant culture and treatment

ATR (99% purity) was obtained from the Institute of Pesticide Science, Academy of Agricultural Sciences in Nanjing, China. Uniform seeds of rice (Japonica cv. Nipponbare) were surface sterilized by 5% H_2_O_2_, rinsed thoroughly with distilled water and germinated for two days on a floating plastic net under a 28 °C dark condition. After germination, seedlings were grown on 1/2-strength modified Hoagland nutrient solution under the condition of a 14/10 light/dark cycle, 30/25 ± 1 °C (day/night) and 200 μmol m^−2^ s^−1^ light intensity. Several days later (depending on experiments), they were used for treatments and analysis. For bisulfite- and RNA-sequencing, plants were treated with 0 (control, CK) and 0.4 mg L^−1^ ATR for 0, 2, 4 and 6 d. After that, roots and shoots were separately harvested and immediately frozen liquid nitrogen. Treatment solutions were renewed every day. For DNA methylation inhibitor experiment, seven day-old seedlings were pretreated with 20 μM 5-azacitidine (5-aza-2-deoxycytidine or AZA) for 16 h and then exposed to 0.4 mg L^−1^ atrazine for 4 d. Studies or experiments were performed in triplicate.

Homozygous mutant Osrdr1 (His background, accession # H0643) was obtained from the rice retrotransposon Tos17 insertion lines (http://tos.nias.affrc.go.jp/). *jmj706* (KT background, accession # PFG_K-0085.R), *Met1* (KT, PFG_K-00237.L), *sdj714* (DJ background, PFG_2A-30024.L), *sdj724* (DJ, PFG_3A-02454.L), Osdrm2 (DJ, PFG_3A-05515.R) and Osros1 (DJ, PFG_1B-00930.R) were obtained from http://signal.salk.edu/cgi-bin/RiceGE. The mutants were characterized using semi and qRT-PCR (Supplementary Fig. S14). The seven day-old mutants and their wild types (WT) were treated with 0 (control, CK) and 0.4 mg L^−1 ^ATR for 4 d. After that, roots and shoots were separately harvested and immediately frozen liquid nitrogen for the subsequent analysis.

### Methyl cytosine-sequencing library construction and genome bisulfite sequencing

Genomic DNAs were isolated from ATR-free and ATR-exposed rice samples using QIAamp DNA Mini Kit (Qiagen Inc., USA). A total of 0.5~1 μg of genomic DNA was used to generate bisulfite sequencing (BS-Seq) libraries. DNA was fragmented by sonication to a mean size of approximately 250 bp. Libraries were constructed using the Illumina Paired-End protocol. Ligated DNA was bisulfite-converted using the EZ DNA Methylation-Gold kit (ZYMO, Qiagen, USA). DNA libraries of different insert size were excised from the same lane of a 2% TAE agarose gel. The bisulfite conversion of rice DNA was carried out using a modified NH_4_HSO_3_-based protocol^57^. Bisulfite-treated DNA was purified by using QIAquick Gel Extraction kit and amplified by PCR. The resultant DNA was applied to paired-end sequencing with the read length of 44 or 75 nt for each end using the ultrahigh-throughput Illumina Genetic Analyzer (GA 2). All reads from the rice samples were mapped to the Nipponbare genome sequence database RGAP (Rice Genome Annotation Project, http://rice.plantbiology.msu.edu/index.shtml). Only reads uniquely mapped to either of the strands (using SOAP 2.21) were retained for further analysis. The base calls per reference position on each strand were used to identify methylated cytosines at 1% the false discovery rate (FDR)^57^. The number of reads supporting methylcytosines was calculated for each genomic feature (5′-UTR, 3′-UTR, CDS, etc.) and sequence context separately and normalized as reads per kilobase of transcript per million reads (RKTM) values. Fisher’s exact test was performed on the read counts of a pair of tissues for each gene, and a gene was defined as differentially methylated when FDR (calculated by the Benjamini-Hochberg method) was less than 0.05 and the ratio of RKTM values was more than 2. Methylation levels were calculated by the ratio of mC/(mC + non-mC). Differentially methylated regions (DMRs) for ATR-treated sample were defined by comparing its methylation levels in each cytosine contexts with its ATR-free sample data.

### RNA high-throughput sequencing and data processing

Total RNA from each sample were extracted from shoots and roots using Trizol (Invitrogen, Carlsbad, CA) and used for creating libraries. The RNA-sequencing was performed using the Illumina^TM^ Hi-seq 2000 system (Illumina, http://www.illumina.com) based on the protocol described previously^12^. Plants were treated in the same way as the method of BS-Seq, but four libraries (shoots and roots with or without ATR treatment) were constructed for RNA-Seq. A rigorous algorithm was used to identify differentially expressed genes between the controls (shoot/root without ATR treatment) and treatments (shoot/root with ATR treatment). The *p*-value corresponds to the differential gene expression test. FDR is a commonly used method to determine the threshold of the *p*-value in multiple tests and analyses and is obtained by manipulating the FDR value. We used FDR ≤ 0.001 and the absolute value of Log_2_Ratio ≥1 as a threshold to judge the significance of gene expression differences. For gene expression profiling, Gene Ontology (GO) enrichment of functional significance was analyzed using the BGI WEGO (Web Gene Ontology Annotation Plot, http://wego.genomics.org.cn/cgi-bin/wego/index.pl).

### Validation of BS-Seq by bisulfite-PCR

DNA (1–500 ng) of validated gene was bisulfite-converted using the EpiTect® Bisulfite kit. The PCR-amplified DNA was cloned into vector pEASY-T1 Simple Cloning Vector (TransGen Biotech, China) and transformed into DH5α cells (TransGen Biotech, China), sequenced by terminal labeling using BigDye Terminator v3.1 (Applied Biosystems, http://www.appliedbiosystems.com/). The sequencing information was analyzed using analysis with BiQ Analyzer. The target sequence was set as the subject and the raw reads as the query and applied blastn to align the reads to the target sequences (*E*-value < 1e-3, matched sequences ≥30). 100% of the reads were successfully mapped to the target regions of gene.

### Transcript analysis by quantitative RT-PCR

Total RNA was extracted from tissues using Trizol (Invitrogen, Carlsbad, CA). One microgram of total RNA was incubated at 37 °C for 30 min with 1 unit of RNase-free DNaseI (Takara) and 1 μL 10 × reaction buffer with MgCl_2_ and incubated with 1 μL of 50 mM EDTA for DnaseI inactivation at 65 °C for 10 min. A 1% agarose gel stained by ethidium bromide was run to indicate the integrity of RNA. The reverse transcription reaction mixture (20 μL) contained 1 μg RNA, 100 μM oligo (dT) primers (1 μL), 10 mM dNTP (deoxyribonucleotide triphosphate) mixture (1 μL), 40 U μL^−1^ of RNase inhibitor (0.5 μL), 200 U μL^−1^ of M-MLV reverse transcriptase (0.5 μL) and 4 μL of 5 × M-MLV buffer. The reaction solution was maintained at 42 °C for 10 min and heated at 95 °C for 2 min. The resultant cDNA was then diluted 5 fold with sterile water and kept at −20 °C for reverse transcription PCR and and quantitative real-time PCR (qRT-PCR) analysis.

qRT-PCR was performed based on the protocol described previously^12^. A reaction mixture for each PCR run was prepared with the SYBR Green PCR Core Reagents (TaKaRa), with a total volume of 25 μL containing 2 μL of template cDNA, 12.5 μL of the 2 × TransStart^TM^ Top Green qPCR SuperMix (Beijing TransGen Biotech Co., Ltd.) and 200 nM primers. The thermal cycling conditions were 1 cycle of 94 °C for 30 s for denaturation and 40 cycles of 94 °C for 5 s and 60 °C for 30 s for annealing and extension. The primers were used listed in Supplementary Table S7.

### Effect of DNA methylation inhibitor on phenotype of rice under ATR exposure

Fresh shoots and roots were weighted and their elongation of fresh individual plants was measured with a ruler. Chlorophyll content was assayed according to the method of extraction with 80% acetone. Plasma membrane permeability of tissues was determined according to the method of Belkhadi *et al.*^58^ with some modification. Leaf and root segments were immersed in tubes with 20 mL distilled water for 30 min, followed by measurement of conductivity of bathing medium (EC1) with a conductivity meter (METTLER TOLEDO FE30-FiveEasy™). Samples were boiled for 20 min and the conductivity of killed tissues (EC2) was measured. The percent leakage of electrolytes was calculated as the ratio of EC1/EC2.

### Chemical analysis of ATR accumulation and metabolites in rice tissues

Atrazine and its metabolites in rice tissues were analyzed based on the method of Lu *et al.*^59^ Fresh tissues (2 g) were ground with liquid nitrogen and ultrasonically extracted three times with mixed acetone−water (3/1, *v*/*v*), followed by centrifugation at 4,000 × g for 10 min and filtration. The filtrate was pooled and concentrated. The residual water was loaded onto an LC-C_18_ solid phase extraction (SPE, Supelclean^TM^, 300 mg) column. Eluates were discarded and the column was washed with 2 mL of methanol−water (80/20, *v/v*). Ten μL internal standard samples (200 mg L^−1^ Ametryn, AME) were added in the methanol solution for internal standard calibration.

Liquid chromatography/mass spectrometer (LC/MS) analysis of rice tissues extracts was performed with an ultra-performance liquid chromatography (UPLC) apparatus equipped with a Waters Acquity PDA detector (Waters, USA) connected to a WATERS SYNAPT SYNAPT Q-TOF Mass Spectrometer (Waters, USA) (UPLC/Q-TOF-MS/MS). Chromatographic operation was taken under conditions as followings: BEH C_18_ columns (2.1 × 50 mm, granulation of 1.7 μm, Waters); injection volume, 2 μL; mobile phase with two solvents: A (100% Acetonitrile) and B (99.9% H_2_O/0.1% formic acid, *v*/*v*) at a 0.3 mL min^−1^ flow rate.

The electrospray (ESI) in positive mode and time-of flight mass spectrometer parameters (TOF-MS) were optimized to reach the best sensibility to ATR and its metabolites ion [M + H]^ + ^for the *m/z* range from 50 to 1,000. The desolvation gas flew at 500 L h^−1^. A capillary voltage was set at 3.5 kV. The cone voltage was set at 30 V. The collision energy was set between 6 eV and 15 eV to achieve high fragmentation of protonated and deprotonated molecules ([M + H]^ + ^). The MS/MS experiments were performed using a collision energy ramp of 8 eV to 15 eV (positive ion mode). Leucine enkephaline at 2 ng μL^−1^ (exact mass of 556.2771 Da [M + H]^ + ^) was infused at a flow rate of 10 μL min^−1^ in the lock spray channel.

## Additional Information

**How to cite this article**: Lu, Y. C. *et al.* Genome-wide identification of DNA methylation provides insights into the association of gene expression in rice exposed to pesticide atrazine. *Sci. Rep.* **6**, 18985; doi: 10.1038/srep18985 (2016).

## Supplementary Material

Supplementary Information

## Figures and Tables

**Table 1:** Methylation levels (Log_2_ fold change ≥ 1) of miRNA loci in ATR-exposed rice.

miRNA	(ATR)	(CK)	Log_2_ML_ATR_/ML_CK_	E	TA	Targets	
						Gene ID	Description
MIR394	0.45	0.21	2.14↑	0.0	21.4	LOC_Os01g69940.1	OsFBX32-F-box domain containing protein
				2.5	20.9	LOC_Os04g40220.1	Transposon protein, putative, CACTA, En/Spm
MIR812c	0.19	0.04	2.25↑	0.0	15.3	LOC_Os03g48380.1	Expressed protein
				0.5	17.2	LOC_Os02g23823.2	Helix-loop-helix DNA-binding domain containing protein,
				1.0	8.2	LOC_Os07g26690.1	Aquaporin protein, putative
				2.5	11.6	LOC_Os04g20474.3	UDP-glucoronosyl /UDP-glucosyl transferase
MIR812o	0.15	0.05	3.00↑	1.0	14.5	LOC_Os04g33720.1	Glycosyl hydrolases, putative
				3.0	11.4	LOC_Os04g20474.1	UDP-glucoronosyl/UDP-glucosyl transferase domain containing protein
MIR812q	0.37	0.17	1.12↑	1.5	14.6	LOC_Os03g22050.1	CAMK includes calcium/calmodulin depedent protein kinases
				3.5	15.7	LOC_Os04g08828.1	Cytochrome P450, putative
MIR812r	0.37	0.17	1.12↑	1.5	12.2	LOC_Os11g09260.1	Expressed protein
				3.5	15.4	LOC_Os12g29700.1	Retrotransposon protein, putative, unclassified
MIR1437a	0.76	0.35	2.17↑	3.0	22.1	LOC_Os07g05420.1	Anthocyanidin 3-O-glucosyltransferase
				3.5	21.2	LOC_Os07g05330.1	Transposon protein, putative, CACTA, En/Spm
				3.5	16.5	LOC_Os03g19290.2	Mitochondrial import inner membrane translocase subunit Tim17
MIR1437b	0.76	0.35	2.17↑	2.5	14.4	LOC_Os05g08410.2	ATP10 protein
				3.0	14.9	LOC_Os05g40720.1	Glycosyltransferase, family 8
				3.0	21.2	LOC_Os06g13740.1	Transposon protein, putative
MIR1862d	0.47	0.18	1.38↑	2.5	18.5	LOC_Os10g40960.1	Oxidoreductase, 2OG-Fe oxygenase family protein
				2.5	15.9	LOC_Os02g39070.1	Vesicle transport protein GOT1B
				3.0	15.9	LOC_Os01g53390.1	Glucosyltransferase
MIR1862e	0.56	0.21	1.42↑	2.0	18.5	LOC_Os02g30730.1	SART-1 family protein
				3.5	6.6	LOC_Os05g03174.1	Glycosyltransferase family 43 protein
				3.5	11.6	LOC_Os04g46980.1	Cis-zeatin O-glucosyltransferase
MIR2101	0.27	0.09	1.58↑	3.0	10.8	LOC_Os01g04650.1	PB1 domain containing protein
				3.0	16.5	LOC_Os03g51440.1	LRR receptor-like protein kinase
MIR2921	0.18	0.01	4.17↑	3.0	16.6	LOC_Os03g12160.1	Leucine-rich repeat family protein
				3.5	10.9	LOC_Os02g36570.1	ABC1 family domain containing protein
MIR5179	0.36	0.15	1.26↑	1.0	24.6	LOC_Os06g49840.2	OsMADS16 - MADS-box family gene with MIKCc type-box,
				3.0	16.3	LOC_Os03g33300.1	Retrotransposon, putative, centromere-specific
MIR5490	0.43	0.21	1.03↑	2.0	15.6	LOC_Os08g36360.1	Retrotransposon protein, putative, Ty1-copia
				2.5	9.8	LOC_Os12g09400.1	Retrotransposon protein, putative, Ty1-copia
				2.5	12.8	LOC_Os07g12350.1	Retrotransposon protein, putative, Ty1-copia
MIR5810	0.87	0.29	3.00↑	3.5	14.8	LOC_Os01g32870.2	Heat shock protein DnaJ
				3.5	22.1	LOC_Os08g01120.1	Sulfate transporter, putative
				3.5	15.8	LOC_Os03g32470.2	Leucoanthocyanidin dioxygenase, putative
MIR5817	0.24	0.10	1.26↑	1.0	11.5	LOC_Os03g64140.1	Expressed protein
				3.0	18.0	LOC_Os10g06230.1	Retrotransposon protein, putative, Ty3-gypsy
				3.5	14.1	LOC_Os08g45010.1	ABC transporter, ATP-binding protein
				3.5	4.8	LOC_Os07g30950.1	Hydroxylase

↑,up-methylation in comparison with control sample; ML, methylated level; E, expectation value; TA, target accessibility-maximum energy to unpair the target site.
