# Supplementary material for: Genome-wide identification of DNA methylation provides insights into the association of gene expression in rice exposed to pesticide atrazine
Source: Sci Rep. 2016 Jan 7;6:18985. doi: 10.1038/srep18985 (PMC4704053; doi:10.1038/srep18985)
Supplement: Supplementary Information [file srep18985-s1.pdf]

Supplementary Information

Title: Genome-wide identification of DNA methylation provides insights into the association of gene expression in rice exposed to pesticide atrazine

Name of authors: Yi Chen Lu[a,b], Sheng Jun Feng[c], Jing Jing Zhang[a,b], Fang Luo[a,b], Shuang Zhang[d], Hong Yang[a*]

Institute: [a]Jiangsu Key Laboratory of Pesticide Science, College of Sciences, Nanjing Agricultural University, Nanjing 210095, China; [b]Key Laboratory of Monitoring and Management of Crop Diseases and Pest Insects, Ministry of Agriculture, Nanjing Agricultural University, Nanjing, China; [c]Department of Biochemistry and Molecular Biology, College of Life Science, Nanjing Agricultural University, Nanjing 210095, China; [d]State key laboratory of food science and technology, Jiangnan University, Wuxi 214122, China

Authors:

Yi Chen Lu, 2013202040@njau.edu.cn

Sheng Jun Feng, 2014216035@njau.edu.cn

Jing Jing Zhang, 2014202046@njau.edu.cn

Fang Luo, 2013111013@njau.edu.cn

Shuang Zhang, shuangzhang@jiangnan.edu.cn

Hong Yang, hongyang@njau.edu.cn

TABLE OF CONTENT : Supplementary Figures S1-14, Supplementary Tables S1-7 and Supplementary Note

• Supplementary Figures S1-14

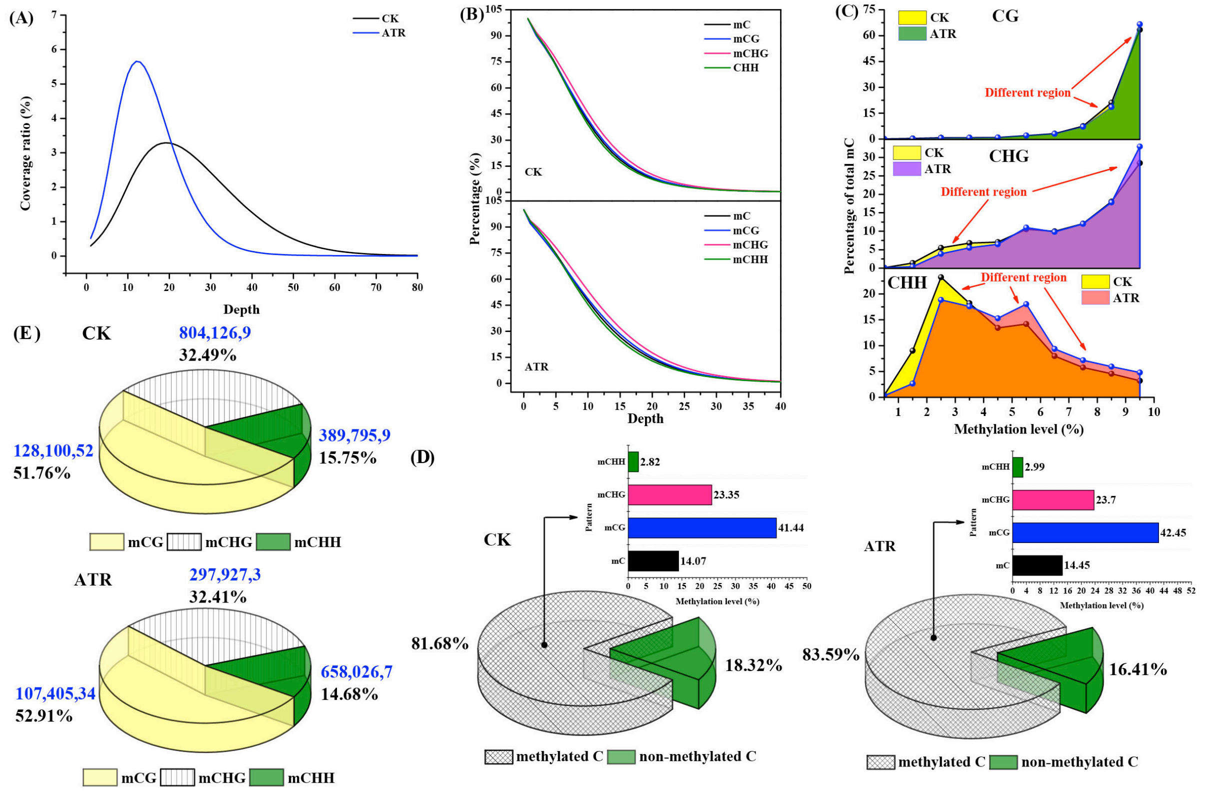

Supplementary Figure S1. Sequencing and mapping summary. (A) The cumulative methylcytosine distribution of effective sequencing depth. The *y* axis indicates the cumulative methylcytosine distribution of CK (control, ATR-free) and ATR-exposed rice under each effective sequencing depth of cytosine (*x* axis). (B) The genomic coverage under different depths of sequencing reads. This figure reflects the overall trend between genomic coverage and sequencing depth. (C) Distribution of the methylation level in each sequence context. The *y* axis indicates the fraction of all methylcytosines that display each methylation level (*x* axis), where methylation level is the ^{m}C/C ratio at each reference cytosine (at least 10 reads required). (D) Levels of methylated cytosines and non-methylated cytosines in whole genome. The bar charts on the top of pie graphs indicate levels of methylated cytosines for each pattern. (E) Percentage of identified methylated cytosines for each sequence context. Blue numbers indicate the number of methylcytosines.

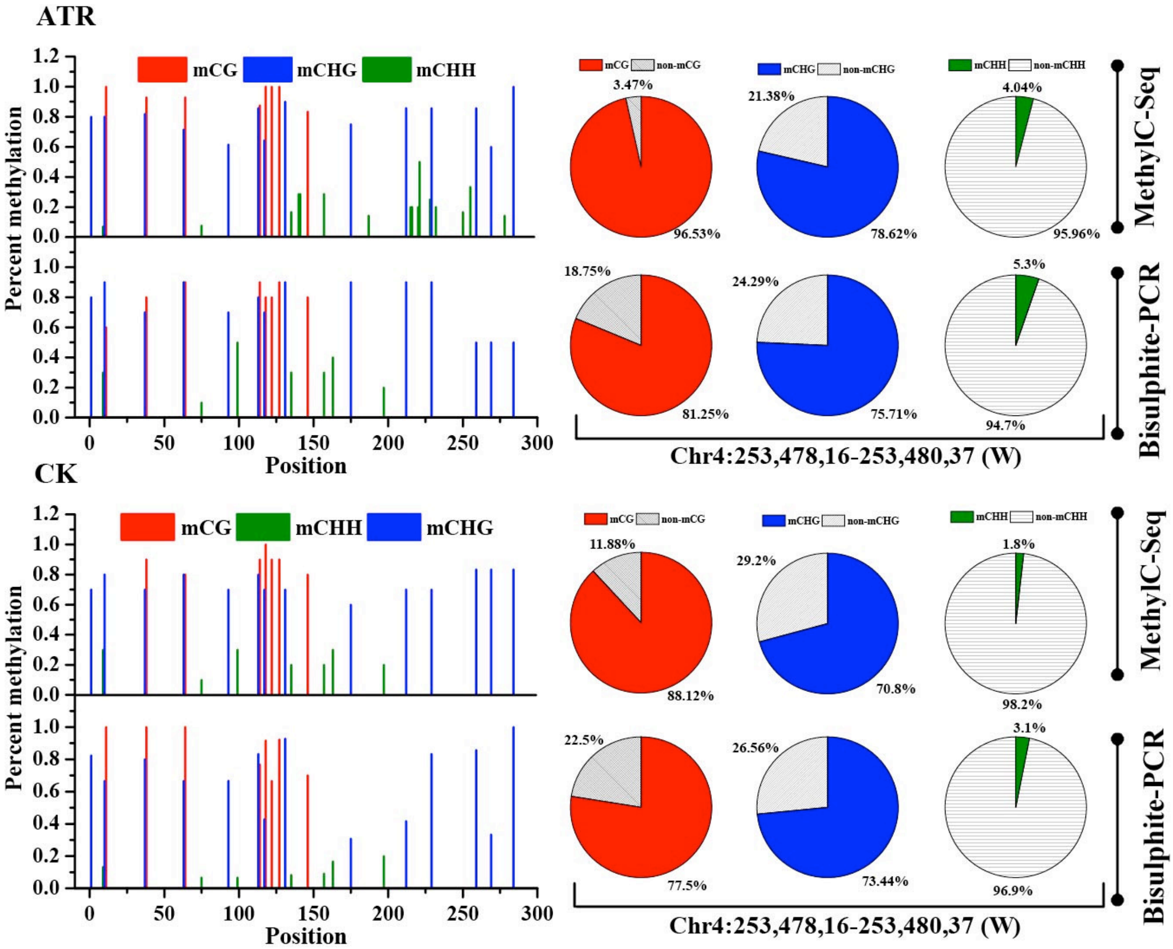

Supplementary Figure S2. Bisulphite-PCR validation of non-CG DNA methylation in an essential detoxification gene LOC_Os10g38470.1.

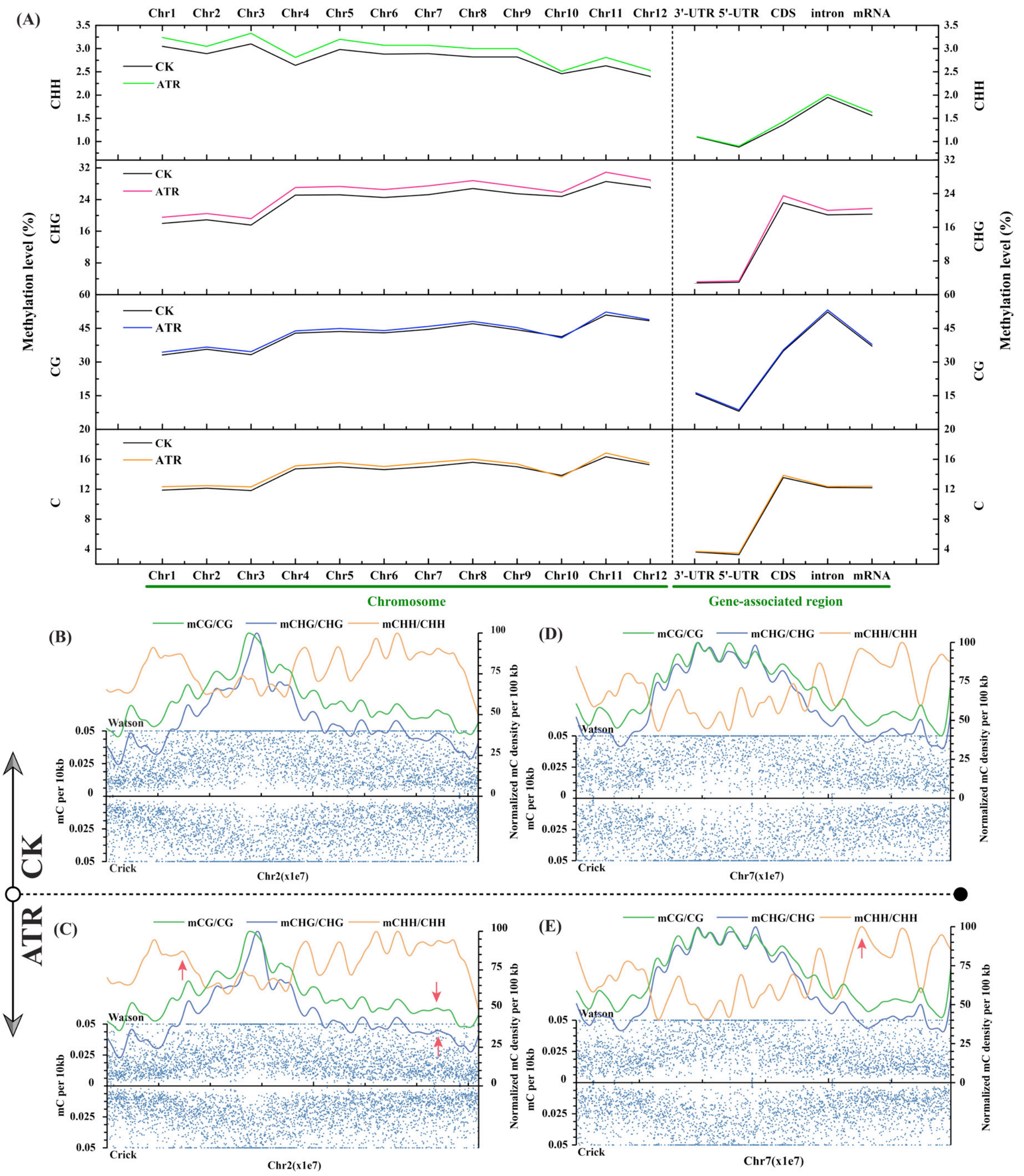

Supplementary Figure S3. The density profiling of methylcytosines in chromosomes and different genomic regions. (A) Levels of CG methylation and non-CG methylation in chromosomes and different genetic regions (3'-UTR, 5'-UTR, CDS, intron and mRNA). (B–E) Blue dots indicate methylcytosine density in CK and ATR-treated tissues in 10-kb windows throughout chromosome 2 and 7. Smoothed lines represent the methylcytosine density in each context in CK and ATR-treated tissues. Red arrows indicate distinct regions of CG and non-CG methylation between the two treatments (B versus C and D versus E).

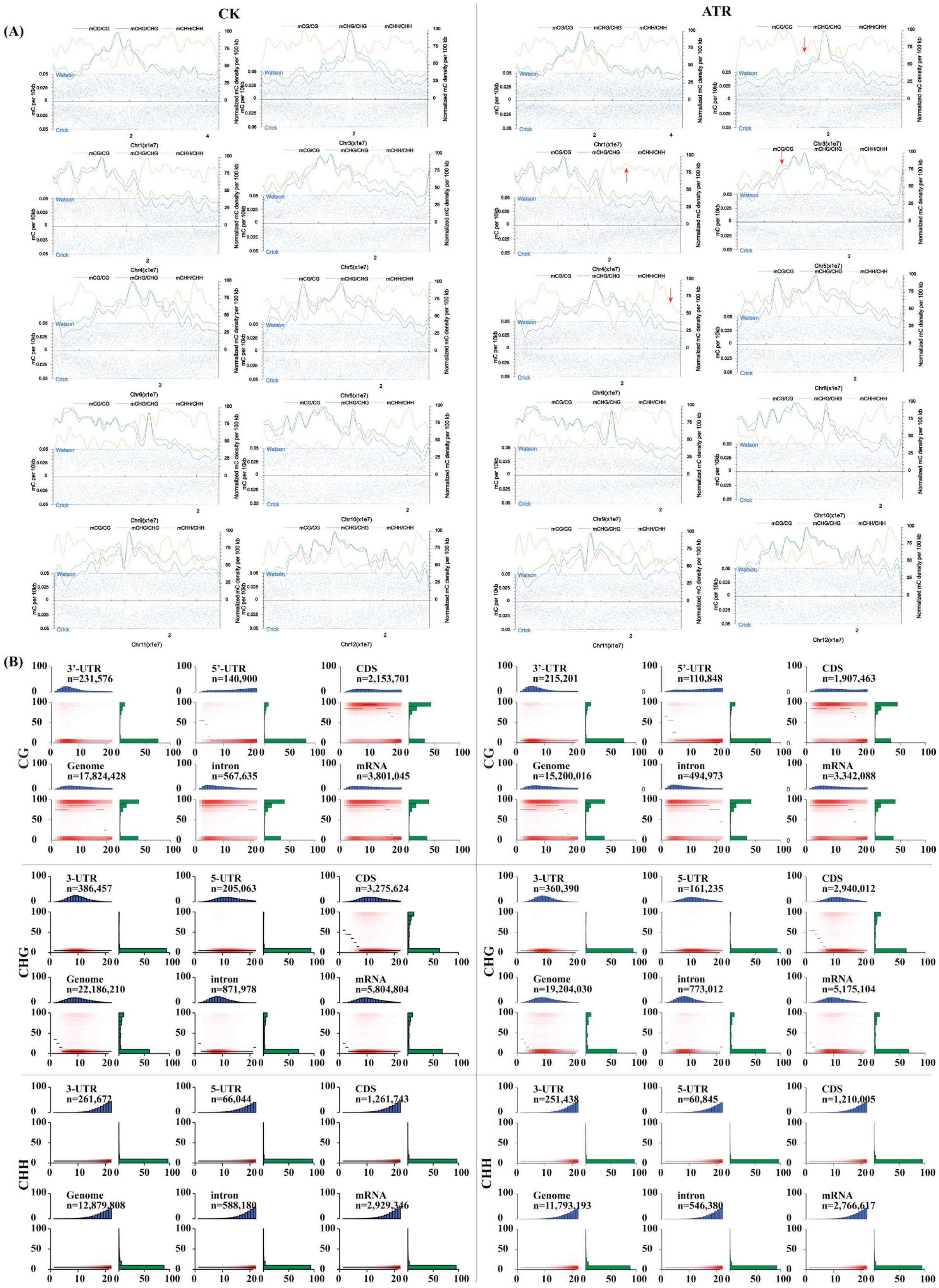

Supplementary Figure S4. (A) The density profile of methylcytosines in chromosomes of rice (Data of chromosome 5 are in Fig. 2A, B). Blue dots indicate methylcytosine density in CK and

ATR-exposed rice in 10 kb windows throughout chromosomes. Smoothed lines represent the methylcytosine (CG、CHG and CHH) density in each context in CK (ATR-free) and ATR-exposed rice. (B) The methylation (CG, CHG and CHH) distributions in different genomic regions. The heatmap of genomic region distribution characteristics displays the number of methylcytosines (4X). The *x* axis indicates the number of methylcytosines in 200 bp windows displaying each mean of methylation levels (*y* axis). Black thread represents the methylated median value in specific density of CHG and CHH. Red zone reflects the particular methylated levels from shallow to deep and the number of methylcytosines. The above blue bar chart represents the density distribution of CHG and CHH and mapping to the horizontal axis. Green column on the right side display the distribution of methylation level. CK: ATR-free.

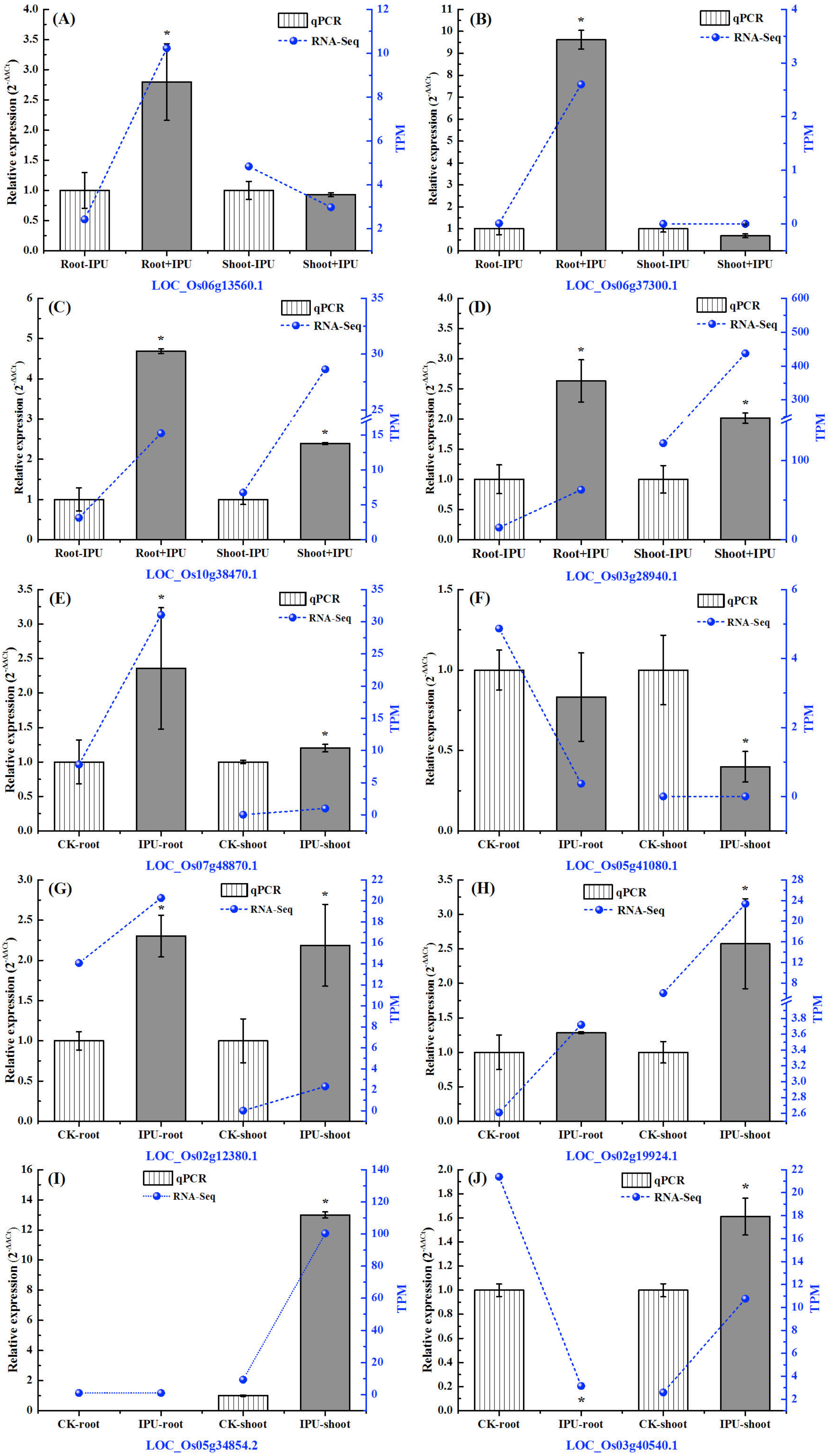
(A)
qPCR
RNA-Seq
Relative expression ($2^{-\Delta\Delta Ct}$)
TPM
Root-IPU
Root+IPU
Shoot-IPU
Shoot+IPU
LOC_Os06g13560.1
(B)
qPCR
RNA-Seq
Relative expression ($2^{-\Delta\Delta Ct}$)
TPM
Root-IPU
Root+IPU
Shoot-IPU
Shoot+IPU
LOC_Os06g37300.1
(C)
qPCR
RNA-Seq
Relative expression ($2^{-\Delta\Delta Ct}$)
TPM
Root-IPU
Root+IPU
Shoot-IPU
Shoot+IPU
LOC_Os10g38470.1
(D)
qPCR
RNA-Seq
Relative expression ($2^{-\Delta\Delta Ct}$)
TPM
Root-IPU
Root+IPU
Shoot-IPU
Shoot+IPU
LOC_Os03g28940.1
(E)
qPCR
RNA-Seq
Relative expression ($2^{-\Delta\Delta Ct}$)
TPM
CK-root
IPU-root
CK-shoot
IPU-shoot
LOC_Os07g48870.1
(F)
qPCR
RNA-Seq
Relative expression ($2^{-\Delta\Delta Ct}$)
TPM
CK-root
IPU-root
CK-shoot
IPU-shoot
LOC_Os05g41080.1
(G)
qPCR
RNA-Seq
Relative expression ($2^{-\Delta\Delta Ct}$)
TPM
CK-root
IPU-root
CK-shoot
IPU-shoot
LOC_Os02g12380.1
(H)
qPCR
RNA-Seq
Relative expression ($2^{-\Delta\Delta Ct}$)
TPM
CK-root
IPU-root
CK-shoot
IPU-shoot
LOC_Os02g19924.1
(I)
qPCR
RNA-Seq
Relative expression ($2^{-\Delta\Delta Ct}$)
TPM
CK-root
IPU-root
CK-shoot
IPU-shoot
LOC_Os05g34854.2
(J)
qPCR
RNA-Seq
Relative expression ($2^{-\Delta\Delta Ct}$)
TPM
CK-root
IPU-root
CK-shoot
IPU-shoot
LOC_Os03g40540.1

Supplementary Figure S5. Quantitative real-time PCR validation of genes which were both |Log_2foldchange|≥1 in differentially expressed genes (DGEs) profiling and ($Log_2 \mu_{treatment}/\mu_{CK} \geq$ 1, *x* axis) indifferenttially methylated valuses. These genes encoding proteins presented as follows. (A) LOC_Os06g13560.1, SAM dependent carboxyl methyltransferase; (B) LOC_Os06g37300.1, cytochrome P450; (C) LOC_Os10g38470.1, glutathione S-transferase; (D) LOC_Os03g28940.1, ZIM domain containing protein; (E) LOC_Os07g48870.1, MYB family transcription factor; (F) LOC_Os05g41080.1, histone H3; (G) LOC_Os02g12380.1, histone deacetylase; (H) LOC_Os02g19924.1, aminotransferase; (I) LOC_Os05g34854.2, gibberellin 20 oxidase 2; (J) LOC_Os03g40540.1, cytochrome P450.

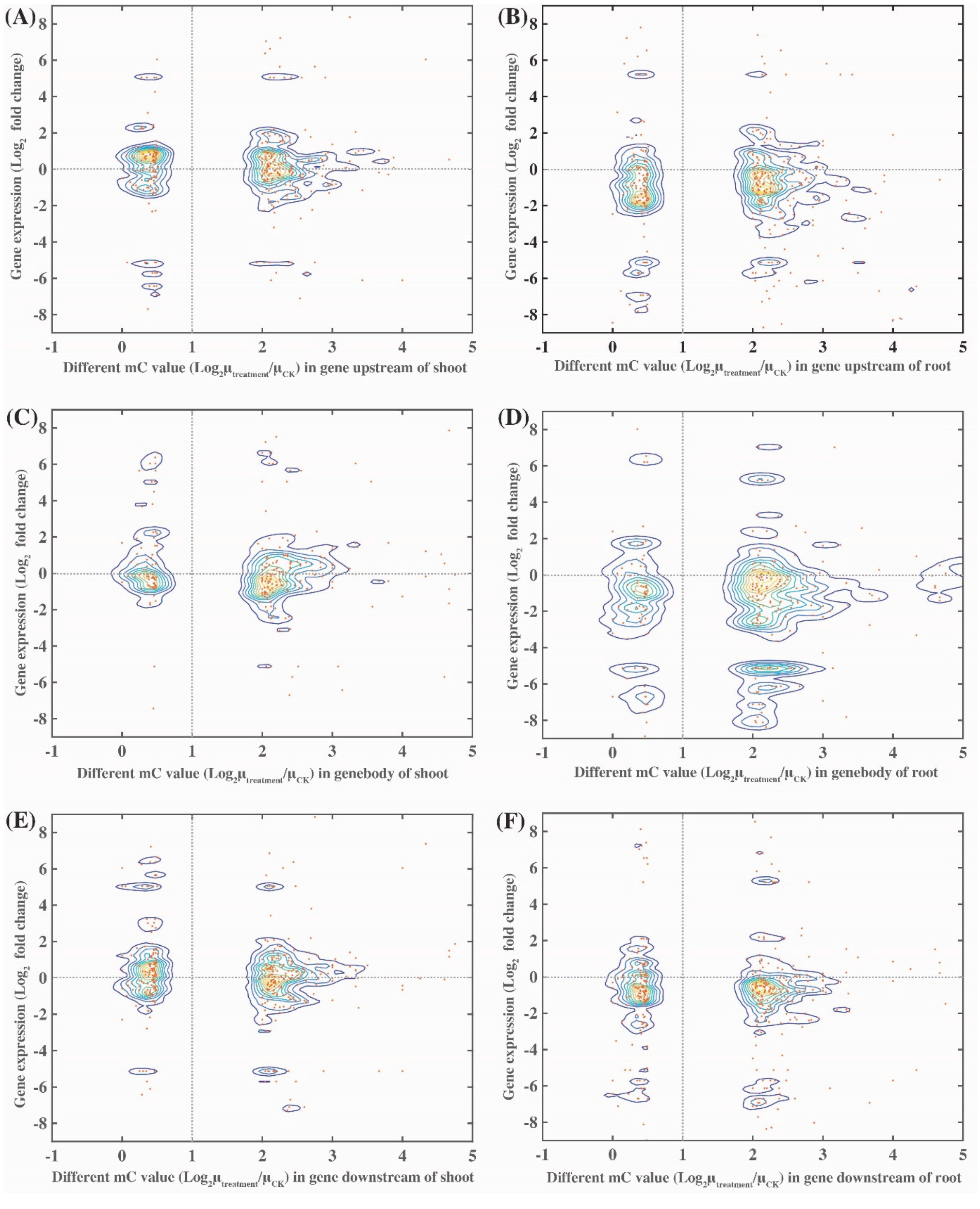

Supplementary Figure S6. Methylation at various genic regions (upstream, genebody and downstream) differentially associated with gene expression. Relative methylation density (*x* axis) as a function of gene expression (*y* axis), with the logarithm value of expression fold change increasing from bottom to top. Colored lines represent data point density.

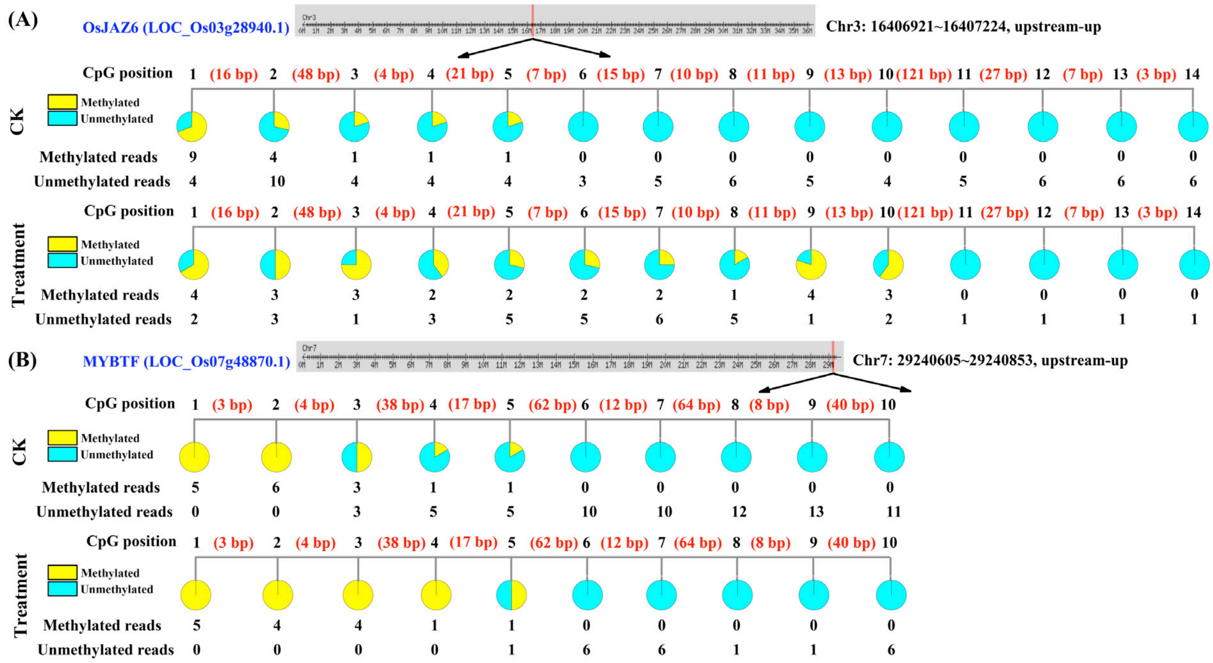

Supplementary Figure S7. Profiling of the DNA methylation marks in the specific regions of genes LOC_Os03g28940.1 (*OsJAZ6*, A) and LOC_Os07g48870.1 (MYB transcription factor, B).

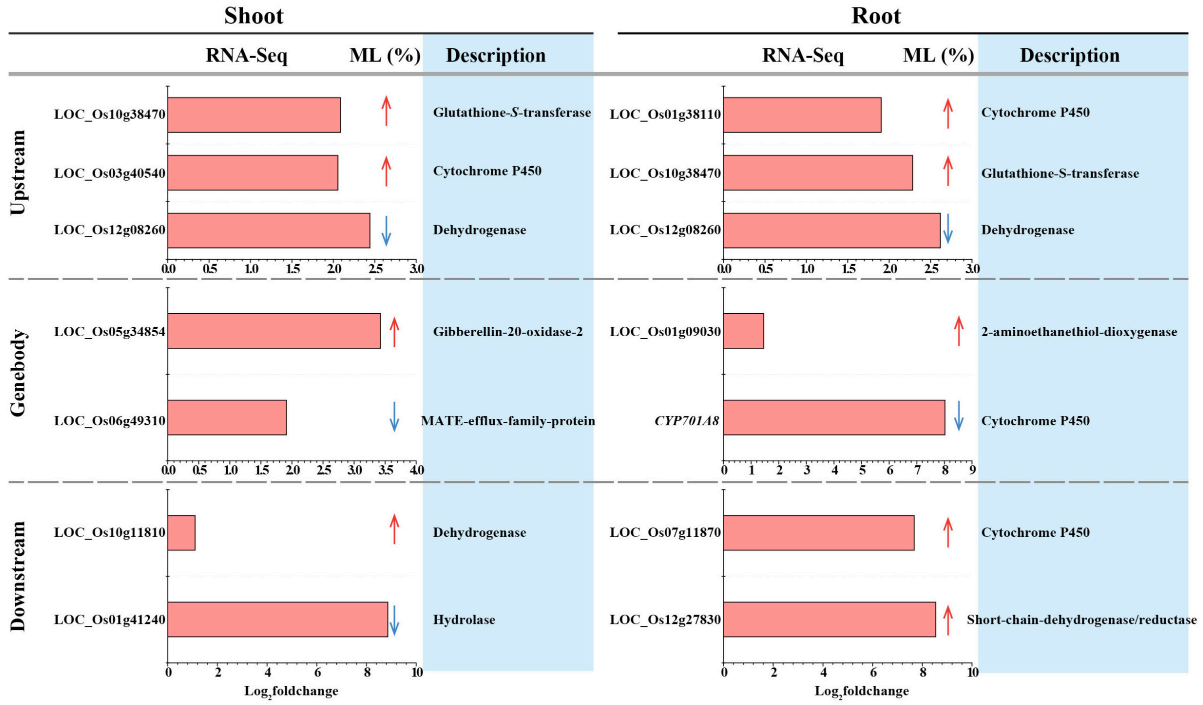

Supplementary Figure S8. The expression and methylation level of ATR metabolism-based genes in shoot and root under ATR exposure. ML, methylation level.

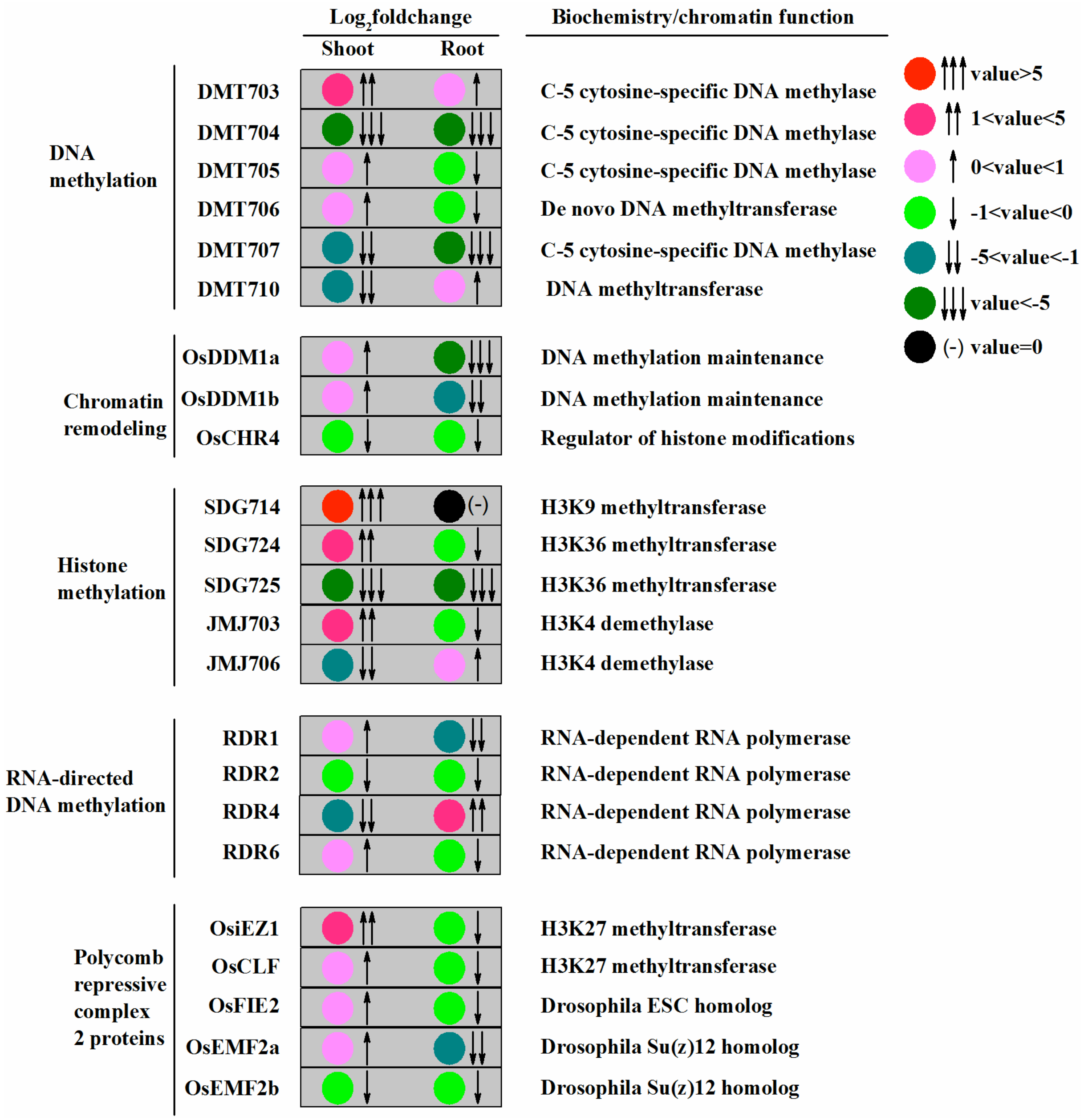

Supplementary Figure S9. Expression of genes associated with DNA methylation under ATR exposure. The data were retrieved from datasets from the high-throughput sequencing of rice exposed to ATR (see Materials and Methods) and are represented as the ratio of Log_2 (TPM_{ATR}/TPM_{CK}).

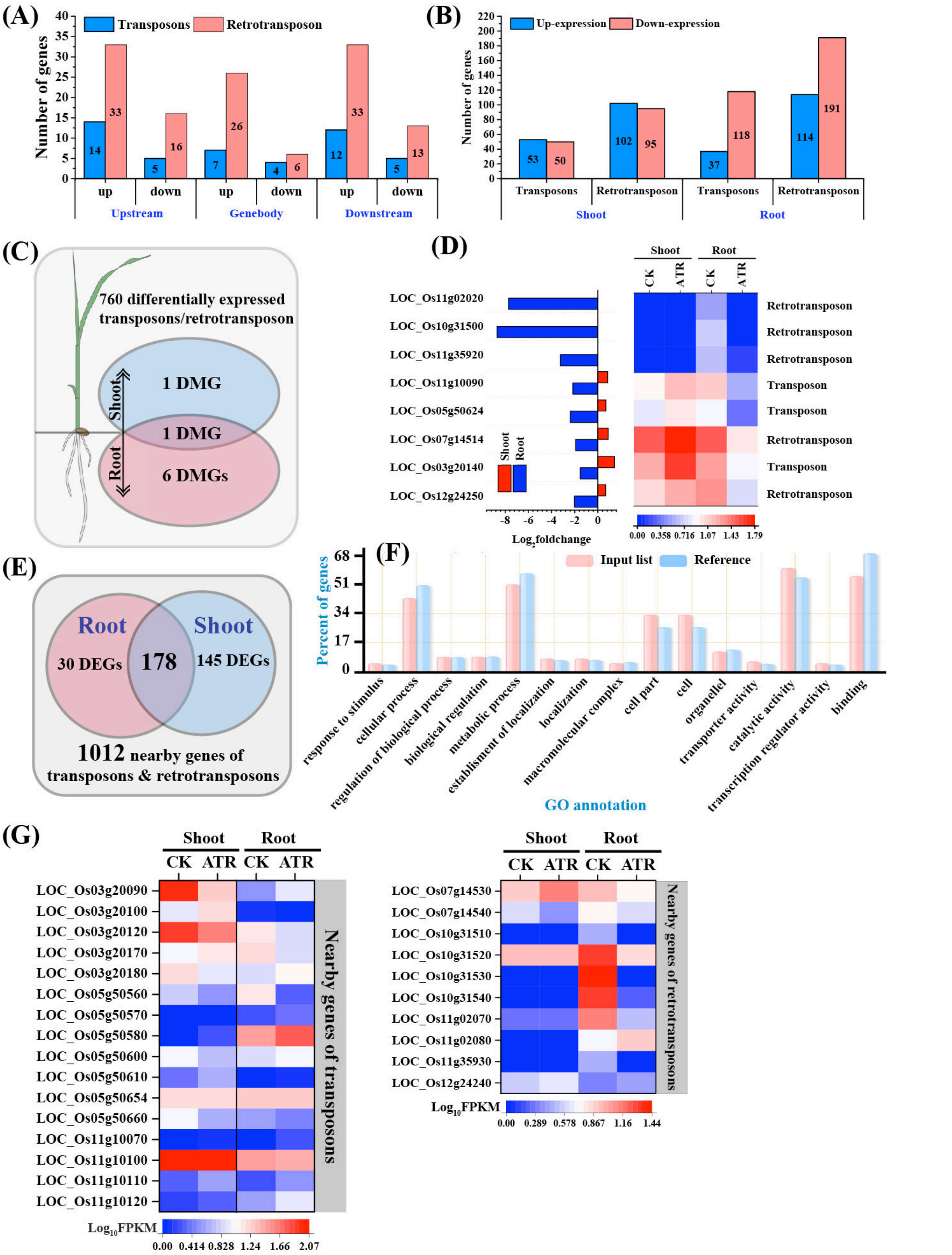

Supplementary Figure S10. The effect of ATR on DNA methylation and expression of transposons and retrotransposons. (A) The numbers of transposons and retrotransposons with

differential DNA methylation levels (≥ 2-fold, $p<0.05$) under ATR stress at various genomic regions. (B) The numbers of transposons and retrotransposons with differential expression (Log_2|fold change|≥ 1, $p<0.05$) under ATR stress in rice shoots and roots. (C) Venn diagrams display the overlap between differentially expressed genes (DEGs) and differentially methylated genes (DMGs) in shoots and roots. (D) The expressions of transposons and retrotransposons with differential expression and methylation level. Data were presented as Log_2foldchange in left chart and log_{10}FPKM in heat map. (E) Venn diagrams indicate the overlap between the genes nearby transposons/retrotransposons and those with differential expression in shoots and roots. The eight genes which are the nearest distance to each transposons and retrotransposons, present as the nearby genes. (F) Go enrichment analysis of 178 DEGs nearby ATR-responsive transposons/retrotransposons. (G) Heat map represents the DEGs nearby the eight transposons/retrotransposons with differential expression and methylation level. Data were presented as log_{10}FPKM in heat map.

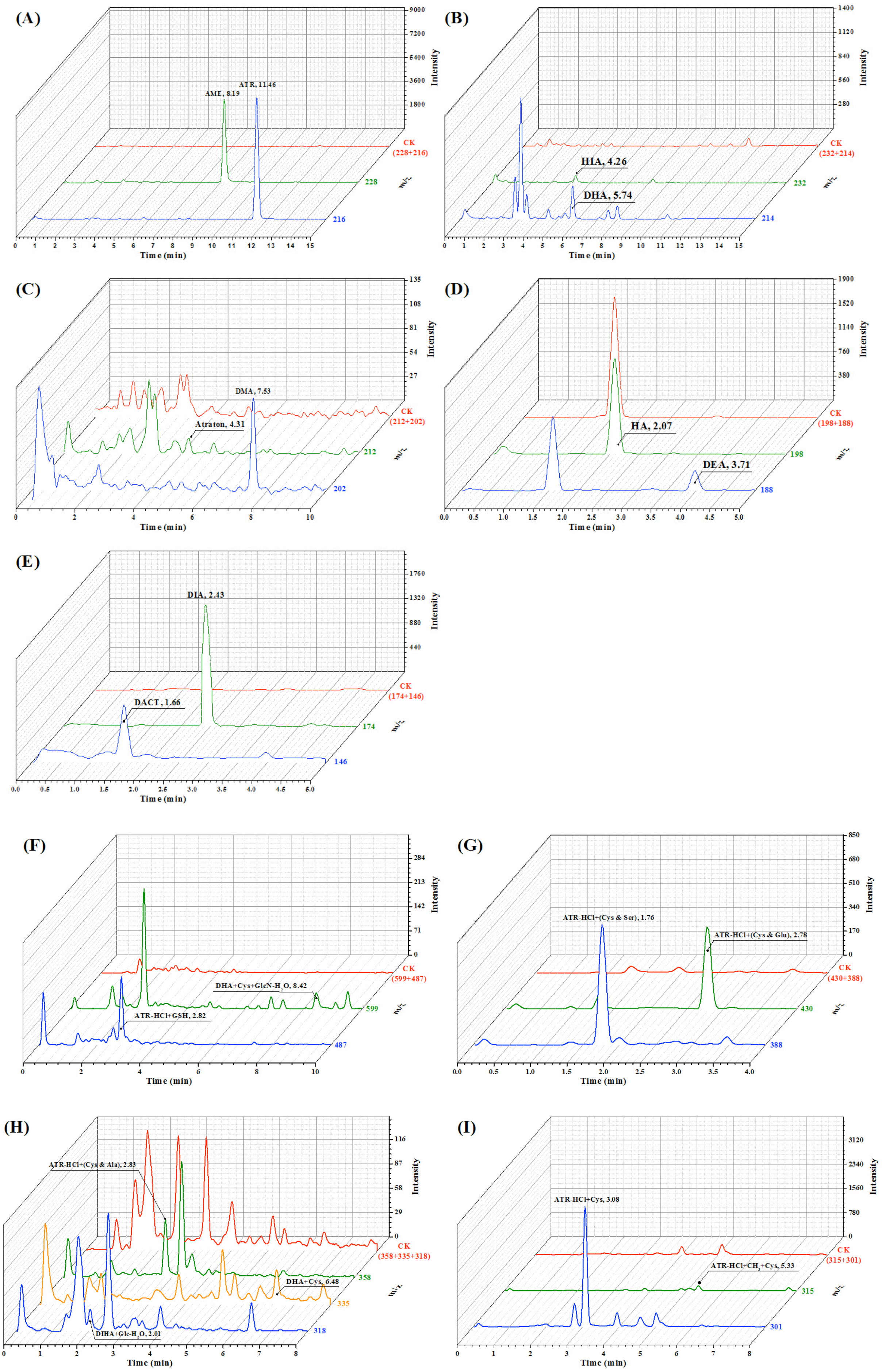

(A)
AME, 8.19
ATR, 11.46
CK (228+216)
228
216
Intensity
m/z
Time (min)
(B)
HIA, 4.26
DHA, 5.74
CK (232+214)
232
214
Intensity
m/z
Time (min)
(C)
DMA, 7.53
Atraton, 4.31
CK (212+202)
212
202
Intensity
m/z
Time (min)
(D)
HA, 2.07
DEA, 3.71
CK (198+188)
198
188
Intensity
m/z
Time (min)
(E)
DIA, 2.43
DACT, 1.66
CK (174+146)
174
146
Intensity
m/z
Time (min)
(F)
DHA+Cys+GlcN-H_2O, 8.42
ATR-HCl+GSH, 2.82
CK (599+487)
599
487
Intensity
m/z
Time (min)
(G)
ATR-HCl+(Cys & Ser), 1.76
ATR-HCl+(Cys & Glu), 2.78
CK (430+388)
430
388
Intensity
m/z
Time (min)
(H)
ATR-HCl+(Cys & Ala), 2.83
DHA+Cys, 6.48
DIHA+Glc-H_2O, 2.01
CK (358+335+318)
358
335
318
Intensity
m/z
Time (min)
(I)
ATR-HCl+Cys, 3.08
ATR-HCl+CH_2+Cys, 5.33
CK (315+301)
315
301
Intensity
m/z
Time (min)

Supplementary Figure S11. UPLC/Q-TOF-MS^2 analysis of atrazine (ATR) and its metabolites and conjugates in rice. The extracted ion chromatograms were obtained from full-scan chromatogram. (A) Extracted ion chromatogram of *m/z* 216.1011 (ATR), *m/z* 228.1286 (AME, internal standard) and their negative control (CK). The metabolites as follow: (B) the peak of *m/z* 232.0955 at 4.26 min and *m/z* 214.0849 at 5.74 min; (C) the peak of *m/z* 212.1499 at 4.31 min and *m/z* 202.0849 at 7.53 min; (D) the peak of *m/z* 198.1342 at 2.07 min and *m/z* 188.0701 at 3.71 min; (E) the peak of *m/z* 174.0538 at 2.43 min and *m/z* 146.0229 at 1.66 min. The conjugates as follow: (F) the peak of *m/z* 599.2006 at 8.42 min and *m/z* 487.2061 at 2.82 min; (G) the peak of *m/z* 430.1852 at 2.78 min and *m/z* 388.1751 at 1.76 min; (H) the peak of *m/z* 358.1642 at 2.83 min, *m/z* 335.1056 at 6.48 min and *m/z* 318.1411 at 2.01 min; (I) the peak of *m/z* 315.1596 at 5.33 min and *m/z* 301.1426 at 3.08 min.

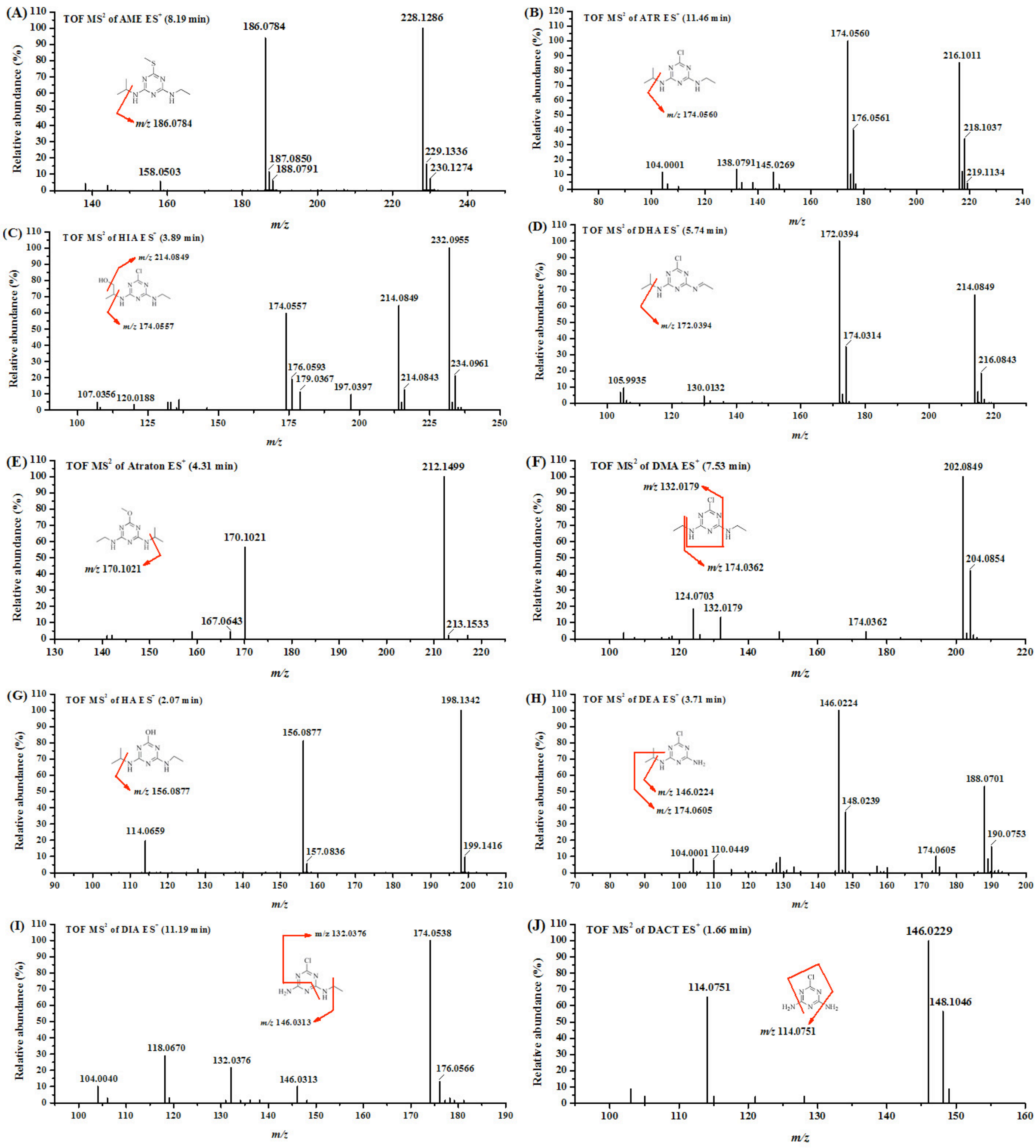

Supplementary Figure S12. Analysis of ATR metabolites. The ion MS^2 spectra were obtained from (A) *m/z* 228.1286 precursor ion, (B) *m/z* 216.1011 precursor ion, (C) *m/z* 232.0955 precursor ion, (D) *m/z* 214.0849 precursor ion, (E) *m/z* 212.1499 precursor ion, (F) *m/z* 202.0849 precursor ion, (G) *m/z* 198.1342 precursor ion, (H) *m/z* 188.0701 precursor ion, (I) *m/z* 174.0538 precursor ion and (J) *m/z* 146.0229 precursor ion of degradation products.

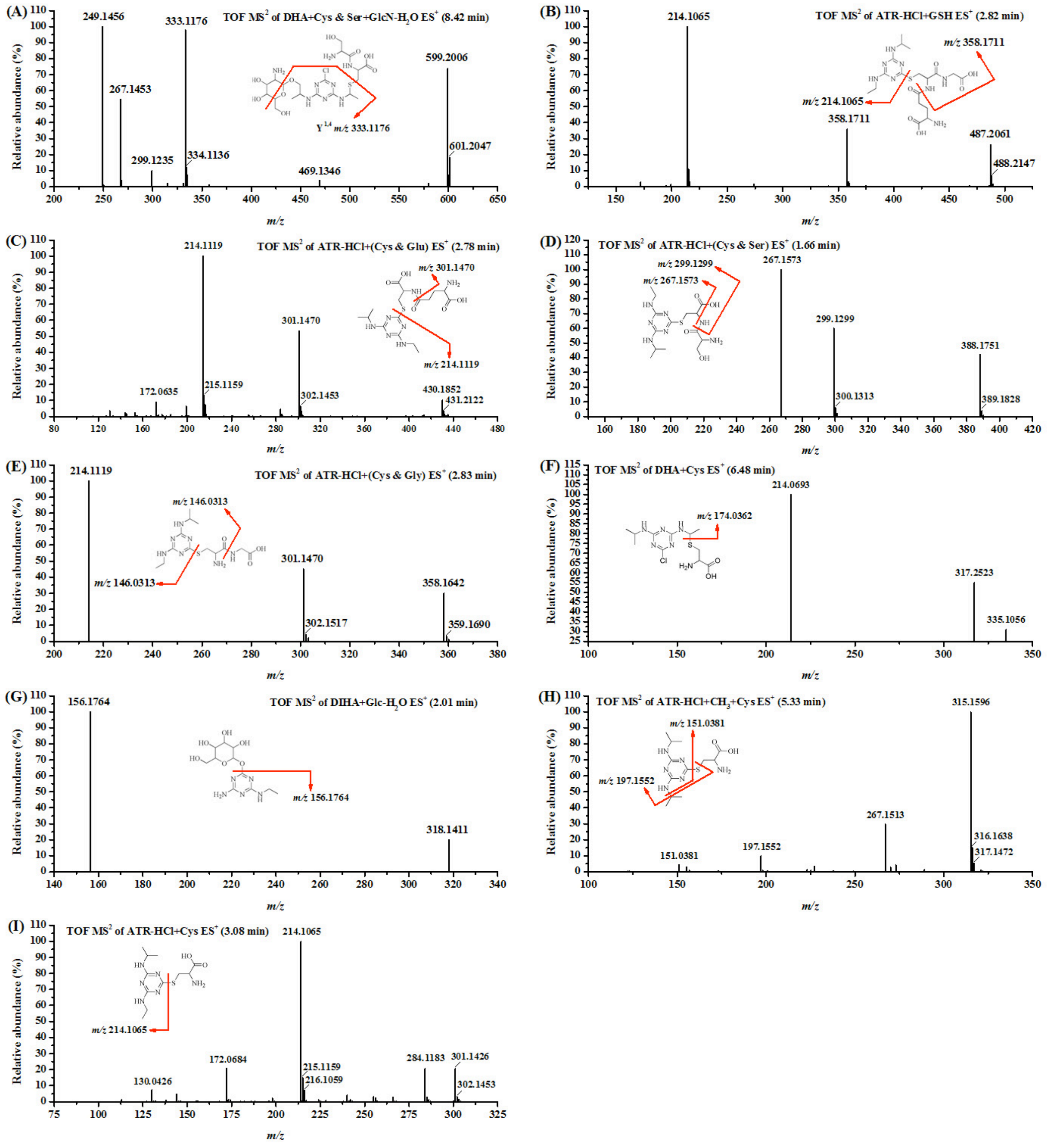

Supplementary Figure S13. Analysis of ATR conjugates. The ion MS^2 spectra were obtained from (A) *m/z* 599.2006 precursor ion, (B) *m/z* 487.2061 precursor ion, (C) *m/z* 430.1852 precursor ion, (D) *m/z* 388.1751 precursor ion, (E) *m/z* 358.1642 precursor ion, (F) *m/z* 335.1056 precursor ion, (G) *m/z* 318.1411 precursor ion, (H) *m/z* 315.1596 precursor ion and (I) *m/z* 301.1426 precursor ion of conjugates.

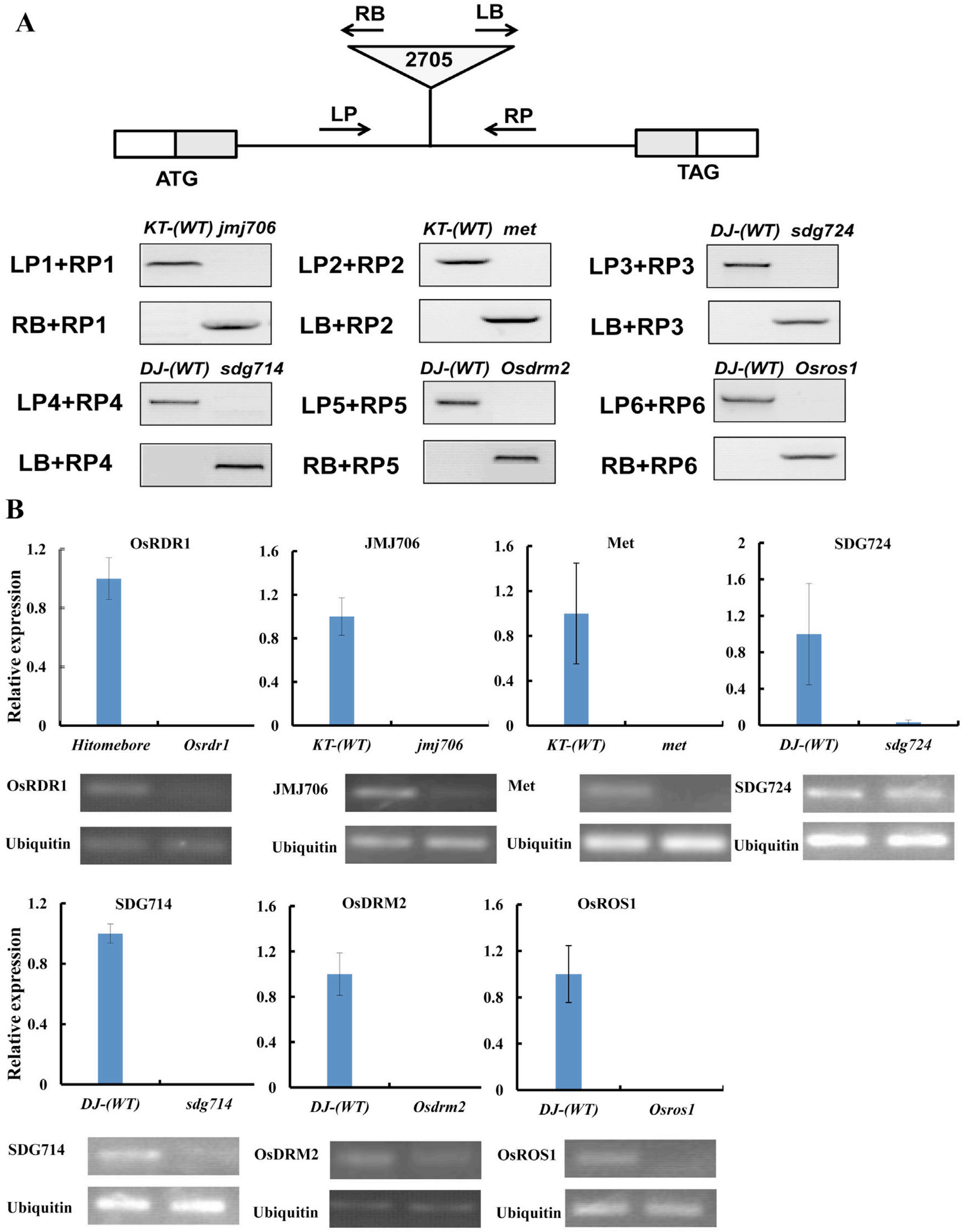

Supplementary Figure S14. Gene expression of the T-DNA insertion mutants. Relative to WT, OsRDR1 (accession # H0643 form the rice retrotransposon Tos17 insertion lines http://tos.nias.affrc.go.jp/), JMJ706 (accession # PFG_K-00085.R form http://signal.salk.edu/cgi-bin/RiceGE), Met (PFG_K-02237.L), SDJ724 (PFG_3A-02454.L), SDJ714 (PFG_2A-30024.L), OsDRM2 (PFG_3A-05515.R) and OsRos1 (PFG_1B-00939.R)

expression was silenced in seedlings of each T-DNA insertion mutants, respectively, as evidenced by both qRT-PCR (top) and semi-quantitative RT-PCR (bottom) amplifications with gene-specific primers. Rice actin and ubiquitin were used as internal reference, respectively.

• Supplementary Tables S1-7

Supplementary Table S1. Output data of BS-Seq

Sample	Library	Insert Size	Conversion rate (%)	Read length (bp)	Clean Reads	Clean data (Gbp)
CK (Control)	WHRICoyhHA AADEAAPEMI-46	332	99.64	90	220,525,514	19.85
ATR	WHRICoyhHA ACDEAAPEMI-47	332	99.66	90	196,667,126	17.7

Supplementary Table S2. Data description of BS-Seq reads for the two rice samples

Sample	Raw reads (M)	Raw data (Gb)	Mapped reads (M)	Mapped data (Gb)	Average map rate (%)	Whole genome average coverage depth (X)
CK (control)	220.53	19.85	115.25	10.37	52.26	27.70
ATR	196.67	17.70	71.39	6.42	36.30	17.16

Supplementary Table S3. The effective coverage of chromosomes and intergenic regions

	Coverage rate (%)								
Fractions		CK				ATR			
		C	CG	CHG	CHH	C	CG	CHG	CHH
Chromosomes	Chr1	94.3	93.47	94.79	94.42	92.28	91.69	93.14	92.23
	Chr2	94.7	93.89	95.25	94.8	92.81	92.19	93.73	92.74
	Chr3	95.45	95.01	96.05	95.41	93.57	93.34	94.55	93.37
	Chr4	91.93	90.67	92.41	92.19	89.77	88.76	90.58	89.86
	Chr5	92.65	91.08	93.11	93.01	90.4	89.02	91.23	90.59
	Chr6	92.57	91.28	92.95	92.85	90.39	89.33	91.13	90.51
	Chr7	92.98	91.78	93.54	93.19	90.91	89.94	91.85	90.96
	Chr8	93.15	92.01	93.6	93.36	90.98	90.1	91.82	91.01
	Chr9	93.26	91.81	93.78	93.55	91.18	89.94	92.02	91.32
	Chr10	92.88	91.64	93.37	93.13	90.72	89.71	91.59	90.79
	Chr11	92.32	91.34	92.99	92.42	90.26	89.49	91.26	90.22
	Chr12	92.64	91.34	93.19	92.87	90.48	89.44	91.37	90.55
Intergenic regions	3-UTR	98.31	98.38	98.74	98.18	97.34	97.69	98.07	97.07
	5-UTR	91.39	93.99	94.75	89.17	86.93	90.09	91.25	84.17
	CDS	92.17	92.43	92.79	91.84	90.13	90.56	91.05	89.63
	intron	95.25	93.47	95.82	95.45	93.57	91.82	94.5	93.68
	mRNA	93.72	93.15	94.33	93.72	91.76	91.26	92.71	91.64

Supplementary Table S4. Distribution of DMRs in chromosome

Chromosome	Number of DMR	Length of DMR	Numbers of DMR	Length of DMR	Numbers of DMR	Length of DMR
	CG		CHG		CHH	
Chr1	187	60661	92	25177	12	2684
Chr2	158	52344	65	17708	14	3098
Chr3	172	55488	82	23336	17	4007
Chr4	147	46910	87	24476	10	2339
Chr5	142	44145	67	18870	13	2874
Chr6	166	51837	75	20611	8	1911
Chr7	158	50445	75	20639	10	2345
Chr8	146	45891	66	18515	10	2245
Chr9	120	37478	66	17541	8	2070
Chr10	98	31942	69	18577	13	3166
Chr11	146	46991	86	23292	12	2736
Chr12	127	42603	91	25055	11	2706
ChrSy	2	699	3	750		
Total	1769	567434	924	254547	138	32181

Supplementary Table S5. 674 genes from the interconnection of DNA methylome (differential DNA methylation levels ≥ 2-fold, $p < 0.05$) and transcriptome (differential genes expression ≥ 2-fold, $p < 0.05$).

Gene ID	Up-methylation			Down-methylation			Gene expression Log_2(+ATR/-ATR)	
	Upstream	Genebody	Downstream	Upstream	Genebody	Downstream	root	shoot
LOC_Os01g02870.1						✔		-1.36
LOC_Os01g04030.1						✔	-1.14	
LOC_Os01g04050.1				✔			1.07	
LOC_Os01g06680.1						✔	-2.49	
LOC_Os01g07940.1	✔						-2.70	
LOC_Os01g09030.1		✔					1.45	
LOC_Os01g11054.2	✔							-2.49
LOC_Os01g11054.3		✔						-2.49
LOC_Os01g11054.4	✔							-2.49
LOC_Os01g11970.1	✔							1.01
LOC_Os01g13120.1						✔		-1.82
LOC_Os01g13210.1						✔	8.12	-1.09
LOC_Os01g13210.2						✔		-1.09
LOC_Os01g24600.1	✔						-1.71	
LOC_Os01g27230.1			✔				-2.23	-1.25
LOC_Os01g32460.1	✔							-1.96
LOC_Os01g32830.1						✔		-2.29
LOC_Os01g32830.2						✔		-2.29
LOC_Os01g33514.1			✔				-1.50	
LOC_Os01g33514.2			✔				-1.50	
LOC_Os01g33514.3			✔				-1.50	
LOC_Os01g34390.1	✔						-1.66	1.83
LOC_Os01g35040.1			✔				2.40	
LOC_Os01g35050.1	✔						-1.86	
LOC_Os01g35050.2	✔						-1.86	
LOC_Os01g35050.3	✔						-1.86	
LOC_Os01g35050.4	✔						-1.86	
LOC_Os01g38100.1		✔			✔		-8.12	
LOC_Os01g38110.1	✔						1.90	
LOC_Os01g38359.1	✔							-2.24
LOC_Os01g38359.2	✔							-2.24
LOC_Os01g39380.1		✔					-3.55	
LOC_Os01g39380.2		✔					-3.55	
LOC_Os01g39380.3		✔					-3.55	

LOC_Os01g41240.1			✔					8.86
LOC_Os01g42650.1		✔					-1.41	
LOC_Os01g42730.1						✔	-1.32	
LOC_Os01g46370.2					✔		-1.76	
LOC_Os01g46500.1						✔	-1.18	1.34
LOC_Os01g46500.2						✔	-1.18	1.34
LOC_Os01g46500.3						✔	-1.18	1.34
LOC_Os01g46500.4						✔	-1.18	1.34
LOC_Os01g46510.1				✔			1.24	
LOC_Os01g46510.2				✔			1.24	
LOC_Os01g48330.1			✔				-1.80	
LOC_Os01g48339.1		✔					-7.82	
LOC_Os01g48444.1	✔						-1.84	
LOC_Os01g50340.1						✔	1.66	
LOC_Os01g51670.1	✔						2.83	
LOC_Os01g52010.1						✔	-3.73	
LOC_Os01g52470.1				✔			7.80	
LOC_Os01g54590.1					✔		-2.74	
LOC_Os01g55940.1		✔					-1.03	10.91
LOC_Os01g56310.1						✔	-2.18	
LOC_Os01g57540.1		✔					-2.15	
LOC_Os01g57550.1	✔						-3.77	
LOC_Os01g57550.2	✔						-3.77	
LOC_Os01g58080.1			✔				-1.12	
LOC_Os01g58080.2			✔				-1.12	
LOC_Os01g58080.3			✔				-1.12	
LOC_Os01g58240.1		✔					-2.66	
LOC_Os01g59730.1	✔						-8.29	
LOC_Os01g59800.1						✔	-1.24	1.28
LOC_Os01g59800.2						✔	-1.24	1.28
LOC_Os01g59800.3						✔	-1.24	1.28
LOC_Os01g60280.1				✔			-1.73	
LOC_Os01g62670.1				✔			1.71	
LOC_Os01g62970.2	✔							1.61
LOC_Os01g63180.1				✔			-1.84	
LOC_Os01g64840.1		✔					-2.82	
LOC_Os01g64920.1				✔			-1.85	
LOC_Os01g65130.1	✔						-1.74	
LOC_Os01g67370.1	✔						-1.57	
LOC_Os01g67370.2	✔						-1.57	
LOC_Os01g67370.3	✔						-1.57	
LOC_Os01g68524.1			✔				-8.03	
LOC_Os01g68524.2			✔				-8.03	
LOC_Os01g68830.1		✔					-3.86	
LOC_Os01g69940.1			✔				-1.24	

LOC_Os01g70720.1				✔			-2.45	
LOC_Os01g70720.2				✔			-2.45	
LOC_Os01g72570.1		✔					2.10	
LOC_Os01g72570.2		✔					2.10	
LOC_Os01g72570.3		✔					2.10	
LOC_Os01g73080.1	✔						-1.42	
LOC_Os01g73080.2	✔						-1.42	
LOC_Os01g73080.3	✔						-1.42	
LOC_Os01g74450.1			✔				-4.49	-2.30
LOC_Os02g01820.1		✔					-7.82	
LOC_Os02g02640.1			✔				-2.03	
LOC_Os02g02720.1						✔		-1.29
LOC_Os02g02720.2						✔		-1.29
LOC_Os02g02720.3						✔		-1.29
LOC_Os02g02720.4						✔		-1.29
LOC_Os02g02720.5						✔		-1.29
LOC_Os02g04160.1						✔		2.85
LOC_Os02g04160.2						✔		2.85
LOC_Os02g04170.1				✔				-2.32
LOC_Os02g04170.3				✔				-2.32
LOC_Os02g04640.1			✔				2.10	
LOC_Os02g07680.5					✔		-2.03	
LOC_Os02g11750.1			✔				-1.28	
LOC_Os02g11750.2			✔				-1.28	
LOC_Os02g11750.3			✔				-1.28	
LOC_Os02g12380.1		✔						7.86
LOC_Os02g12380.2		✔						7.86
LOC_Os02g12380.3		✔						7.86
LOC_Os02g12480.1				✔			-1.54	-1.00
LOC_Os02g12480.10				✔			-1.54	-1.00
LOC_Os02g12480.2				✔			-1.54	-1.00
LOC_Os02g12480.3				✔			-1.54	-1.00
LOC_Os02g12480.4				✔			-1.54	-1.00
LOC_Os02g12480.5				✔			-1.54	-1.00
LOC_Os02g12480.6				✔			-1.54	-1.00
LOC_Os02g12480.7				✔			-1.54	-1.00
LOC_Os02g12480.8				✔			-1.54	-1.00
LOC_Os02g12480.9				✔			-1.54	-1.00
LOC_Os02g13150.1	✔						-1.78	
LOC_Os02g13380.1			✔				1.07	
LOC_Os02g13710.1	✔						-4.79	
LOC_Os02g15810.1			✔				-2.65	
LOC_Os02g16909.1	✔							2.11
LOC_Os02g16909.2	✔							2.11
LOC_Os02g19924.1		✔						1.95

LOC_Os02g20860.1			✔				-1.52	
LOC_Os02g20860.2			✔				-1.52	
LOC_Os02g20870.1	✔						-3.56	
LOC_Os02g20870.2	✔						-3.56	
LOC_Os02g27470.1			✔				-1.31	
LOC_Os02g27594.1			✔				-2.55	-1.15
LOC_Os02g27594.2			✔				-2.55	-1.15
LOC_Os02g27594.3			✔				-2.55	-1.15
LOC_Os02g27950.1				✔			-1.43	
LOC_Os02g27950.2				✔			-1.43	
LOC_Os02g32520.1					✔			2.19
LOC_Os02g32520.2				✔				2.19
LOC_Os02g36414.1					✔		1.07	
LOC_Os02g36619.1						✔	-1.51	
LOC_Os02g37880.1				✔			-2.46	
LOC_Os02g37880.2				✔			-2.46	
LOC_Os02g37880.3				✔			-2.46	
LOC_Os02g38740.1			✔					-1.92
LOC_Os02g41904.1	✔						-4.36	
LOC_Os02g42200.1		✔						-1.85
LOC_Os02g43290.1	✔						-2.65	
LOC_Os02g45600.1						✔	-1.31	
LOC_Os02g45660.1			✔				1.53	
LOC_Os02g49620.1	✔						-1.93	
LOC_Os02g49992.1						✔	-2.08	
LOC_Os02g49992.2						✔	-2.08	
LOC_Os02g49992.3						✔	-2.08	
LOC_Os02g50100.1						✔		-1.25
LOC_Os02g50960.1				✔			-1.71	
LOC_Os02g50960.2				✔			-1.71	
LOC_Os02g51150.1			✔					1.21
LOC_Os02g51150.2			✔					1.21
LOC_Os02g51590.1		✔					-2.81	
LOC_Os02g52280.1	✔						-1.97	
LOC_Os02g52420.1			✔					-1.09
LOC_Os02g52420.2			✔					-1.09
LOC_Os02g52420.3			✔					-1.09
LOC_Os02g55010.1				✔			-1.82	
LOC_Os02g56840.1	✔							-1.61
LOC_Os02g57120.1				✔				2.31
LOC_Os02g57650.1					✔			1.07
LOC_Os02g58480.1		✔					-5.06	
LOC_Os03g02530.1	✔						-3.62	
LOC_Os03g02720.1	✔						-1.71	
LOC_Os03g03410.1		✔					-1.16	

LOC_Os03g04490.1	✔						-2.17	
LOC_Os03g05160.1						✔		1.38
LOC_Os03g07170.1	✔						2.14	
LOC_Os03g09980.1	✔						-11.74	
LOC_Os03g10180.1				✔				1.11
LOC_Os03g12350.1	✔							1.61
LOC_Os03g12350.2	✔							1.61
LOC_Os03g12350.4	✔							1.61
LOC_Os03g12820.1						✔	1.66	2.75
LOC_Os03g12820.2						✔	1.66	2.75
LOC_Os03g14150.1				✔			-1.35	
LOC_Os03g14300.1			✔				2.13	
LOC_Os03g15220.1				✔			-7.82	
LOC_Os03g15580.1		✔					-2.65	
LOC_Os03g17220.1						✔	-1.87	
LOC_Os03g18980.1			✔				-3.29	
LOC_Os03g19400.1	✔							1.56
LOC_Os03g19720.1	✔						2.07	-1.36
LOC_Os03g19940.1	✔							1.31
LOC_Os03g20140.1		✔					-1.53	1.43
LOC_Os03g20860.1			✔				-1.44	
LOC_Os03g22950.1			✔				-2.27	
LOC_Os03g24180.1	✔						-2.40	1.01
LOC_Os03g25750.1		✔						1.41
LOC_Os03g26870.1	✔						-4.76	
LOC_Os03g26870.2	✔						-4.76	
LOC_Os03g27250.1	✔							2.02
LOC_Os03g28940.1	✔						2.04	1.84
LOC_Os03g28940.2	✔						2.04	1.84
LOC_Os03g38000.1		✔					-1.69	
LOC_Os03g38000.2		✔					-1.69	
LOC_Os03g40540.1	✔						-2.76	2.05
LOC_Os03g42464.1	✔						-3.07	
LOC_Os03g44560.1					✔		-3.51	
LOC_Os03g47120.1	✔						-2.13	
LOC_Os03g47610.1	✔							-1.10
LOC_Os03g47610.2	✔							-1.10
LOC_Os03g47610.3	✔							-1.10
LOC_Os03g47610.4	✔							-1.10
LOC_Os03g47610.5	✔							-1.10
LOC_Os03g47610.6	✔							-1.10
LOC_Os03g47610.8	✔							-1.10
LOC_Os03g47930.1						✔	-3.81	
LOC_Os03g48170.1	✔							1.37
LOC_Os03g49430.1	✔							8.37

LOC_Os03g49430.3	✔							8.37
LOC_Os03g49430.4	✔							8.37
LOC_Os03g49640.1	✔						-1.04	
LOC_Os03g49640.2	✔						-1.04	
LOC_Os03g51230.2	✔						-2.81	
LOC_Os03g55180.1	✔						1.68	
LOC_Os03g55950.1	✔						-1.82	
LOC_Os03g55950.2	✔						-1.82	
LOC_Os03g55950.3	✔						-1.82	
LOC_Os03g55950.4	✔						-1.82	
LOC_Os03g56190.1				✔			-1.12	
LOC_Os03g56300.1	✔						-1.40	
LOC_Os03g56820.1		✔					-2.68	
LOC_Os03g56820.2		✔					-2.68	
LOC_Os03g56820.3		✔					-2.68	
LOC_Os03g57050.1	✔							-7.81
LOC_Os03g58070.1					✔		1.77	1.03
LOC_Os03g62180.1		✔					1.50	
LOC_Os03g62180.2		✔					1.50	
LOC_Os03g62180.3		✔					1.50	
LOC_Os03g63730.1			✔					1.04
LOC_Os03g63870.1		✔						-1.05
LOC_Os03g63870.2		✔						-1.05
LOC_Os03g63870.3		✔						-1.05
LOC_Os03g64060.1						✔		1.88
LOC_Os04g01540.1						✔	-1.07	
LOC_Os04g03870.1				✔			-1.38	
LOC_Os04g04390.1						✔	-8.98	
LOC_Os04g09900.1					✔		1.86	
LOC_Os04g09900.2					✔		1.86	
LOC_Os04g09900.3					✔		1.86	
LOC_Os04g15690.1					✔		-1.28	
LOC_Os04g20230.1	✔						-1.27	
LOC_Os04g21110.1		✔					-1.40	
LOC_Os04g25360.1			✔					1.17
LOC_Os04g25360.2			✔					1.17
LOC_Os04g28990.1					✔		-2.65	
LOC_Os04g34984.1					✔		-1.05	
LOC_Os04g34984.2					✔		-1.05	
LOC_Os04g34984.3					✔		-1.05	
LOC_Os04g35130.1			✔					1.34
LOC_Os04g35200.1			✔					1.03
LOC_Os04g36050.1				✔				1.21
LOC_Os04g39600.1	✔						-5.45	
LOC_Os04g40190.1		✔					1.08	

LOC_Os04g40190.2		✔						1.08	
LOC_Os04g40620.1							✔	-8.12	
LOC_Os04g41340.1							✔	-2.61	
LOC_Os04g41850.1	✔							-1.49	
LOC_Os04g41950.1	✔							-1.74	
LOC_Os04g42840.1	✔							-2.10	2.11
LOC_Os04g43490.1			✔					-2.46	
LOC_Os04g43490.2			✔					-2.46	
LOC_Os04g43760.1							✔	-1.13	
LOC_Os04g43760.2							✔	-1.13	
LOC_Os04g43800.1		✔							-1.59
LOC_Os04g44780.1			✔					-8.93	
LOC_Os04g44780.2			✔					-8.93	
LOC_Os04g44850.1			✔					-2.36	
LOC_Os04g47420.1							✔		-2.79
LOC_Os04g49370.1					✔				3.10
LOC_Os04g50720.1		✔						-1.24	1.63
LOC_Os04g52020.1		✔						-2.22	
LOC_Os04g55410.1					✔				1.57
LOC_Os04g56070.1	✔							-1.03	
LOC_Os04g56070.2	✔							-1.03	
LOC_Os04g57700.1			✔					-2.00	
LOC_Os04g57700.2			✔					-2.00	
LOC_Os04g57700.3			✔					-2.00	
LOC_Os04g57700.4			✔					-2.00	
LOC_Os04g58240.1			✔					-1.34	
LOC_Os05g02310.1			✔					-2.46	
LOC_Os05g02740.1	✔							-1.15	
LOC_Os05g03040.1					✔			-2.09	
LOC_Os05g03040.2					✔			-2.09	
LOC_Os05g03040.3					✔			-2.09	
LOC_Os05g03610.1		✔						-3.08	
LOC_Os05g03640.1					✔			1.89	
LOC_Os05g05320.1						✔		-1.57	
LOC_Os05g05320.2					✔			-1.57	
LOC_Os05g10690.1					✔			-1.42	-2.28
LOC_Os05g16740.1		✔				✔		-1.53	
LOC_Os05g24000.1	✔							-1.74	
LOC_Os05g24000.2	✔							-1.74	
LOC_Os05g32110.1	✔							-3.32	
LOC_Os05g32110.2	✔							-3.32	
LOC_Os05g32110.3	✔							-3.32	
LOC_Os05g32110.4	✔							-3.32	
LOC_Os05g33110.1							✔	-1.35	
LOC_Os05g33110.2							✔	-1.35	

LOC_Os05g33110.3						✔	-1.35	
LOC_Os05g33570.1		✔					3.23	1.80
LOC_Os05g34854.1		✔						3.43
LOC_Os05g34854.2	✔							3.43
LOC_Os05g37910.1		✔					-1.66	1.44
LOC_Os05g37910.2	✔						-1.66	1.44
LOC_Os05g38550.1			✔				2.18	1.43
LOC_Os05g38550.2			✔				2.18	1.43
LOC_Os05g38960.1	✔						-8.21	
LOC_Os05g39530.1			✔					1.60
LOC_Os05g39530.2			✔					1.60
LOC_Os05g39540.1	✔						-4.32	
LOC_Os05g39540.2	✔						-4.32	
LOC_Os05g40050.1				✔			-3.01	
LOC_Os05g40050.2				✔			-3.01	
LOC_Os05g41080.1				✔			-3.72	
LOC_Os05g41080.2				✔			-3.72	
LOC_Os05g42220.1			✔				-2.98	
LOC_Os05g42230.1	✔						-1.42	
LOC_Os05g43310.1	✔							-1.65
LOC_Os05g43450.1	✔						-1.76	
LOC_Os05g44100.1		✔					-2.17	
LOC_Os05g44400.1	✔						-3.31	
LOC_Os05g45050.1						✔	-2.94	
LOC_Os05g45050.2						✔	-2.94	
LOC_Os05g45770.1			✔					-2.91
LOC_Os05g45770.2			✔					-2.91
LOC_Os05g46370.1			✔				-8.29	
LOC_Os05g50550.1		✔					-1.71	1.47
LOC_Os05g50624.1		✔					-2.40	
LOC_Os05g50624.2			✔				-2.40	
LOC_Os05g50970.1			✔				-3.05	
LOC_Os05g50980.1	✔						-2.23	
LOC_Os06g02028.1			✔					-1.25
LOC_Os06g02220.1	✔						-1.68	-1.31
LOC_Os06g04190.2	✔						-3.08	
LOC_Os06g05800.1		✔					-1.80	
LOC_Os06g05800.2		✔					-1.80	
LOC_Os06g07830.1	✔						1.26	
LOC_Os06g09340.1						✔	-5.02	
LOC_Os06g09990.1								
LOC_Os06g11990.1		✔					-1.75	
LOC_Os06g12630.1				✔				-1.89
LOC_Os06g12630.2					✔			-1.89
LOC_Os06g12630.3				✔				-1.89

LOC_Os06g12660.1	✔								-1.21
LOC_Os06g13560.1			✔					2.07	
LOC_Os06g13560.2			✔					2.07	
LOC_Os06g13560.3			✔					2.07	
LOC_Os06g13560.4			✔					2.07	
LOC_Os06g13560.5			✔					2.07	
LOC_Os06g13860.1			✔						1.06
LOC_Os06g13860.2			✔						1.06
LOC_Os06g14490.1				✔				-1.30	
LOC_Os06g14490.2				✔				-1.30	
LOC_Os06g15400.1			✔						-1.47
LOC_Os06g16030.1		✔						-1.72	
LOC_Os06g20090.1	✔							-1.37	
LOC_Os06g21140.1	✔							-2.72	
LOC_Os06g23770.1				✔				-1.98	
LOC_Os06g24594.2	✔							-2.42	
LOC_Os06g24594.5	✔							-2.42	
LOC_Os06g24594.6	✔							-2.42	
LOC_Os06g24711.1		✔						1.39	
LOC_Os06g30060.1					✔			-2.38	
LOC_Os06g30060.2					✔			-2.38	
LOC_Os06g33390.1			✔					-1.61	
LOC_Os06g36770.1			✔						1.03
LOC_Os06g36770.2			✔						1.03
LOC_Os06g36770.3			✔						1.03
LOC_Os06g36840.1			✔						-1.79
LOC_Os06g37300.1					✔			8.02	
LOC_Os06g37300.2					✔			8.02	
LOC_Os06g37364.1		✔						-1.67	
LOC_Os06g37364.2		✔						-1.67	
LOC_Os06g37364.3		✔						-1.67	
LOC_Os06g38950.1			✔					-2.03	
LOC_Os06g39640.1		✔						-2.69	
LOC_Os06g41120.1				✔					1.19
LOC_Os06g43430.1		✔						-1.72	
LOC_Os06g46140.1	✔							2.18	1.97
LOC_Os06g47750.1			✔					-3.77	
LOC_Os06g49310.1					✔			-1.93	1.91
LOC_Os06g49310.2					✔			-1.93	1.91
LOC_Os06g49320.1				✔				-1.23	
LOC_Os06g50930.1						✔			6.24
LOC_Os06g50930.2						✔			6.24
LOC_Os07g01550.1		✔							-1.05
LOC_Os07g05360.1						✔			3.03
LOC_Os07g05360.2						✔			3.03

LOC_Os07g05365.1					✔			4.49
LOC_Os07g06660.1			✔					1.02
LOC_Os07g06660.2			✔					1.02
LOC_Os07g08030.1			✔					-1.36
LOC_Os07g08300.1			✔					1.27
LOC_Os07g08300.2			✔					1.27
LOC_Os07g08300.3			✔					1.27
LOC_Os07g08300.4			✔					1.27
LOC_Os07g08300.5			✔					1.27
LOC_Os07g09010.1						✔		3.01
LOC_Os07g09150.1			✔					1.06
LOC_Os07g09914.1			✔				-10.21	
LOC_Os07g11870.1			✔				7.68	
LOC_Os07g13770.1	✔							-2.71
LOC_Os07g14514.2		✔					-1.93	
LOC_Os07g14514.4		✔					-1.93	
LOC_Os07g14514.6		✔					-1.93	
LOC_Os07g14850.1	✔						-1.66	
LOC_Os07g16970.1	✔							2.19
LOC_Os07g16970.2	✔							2.19
LOC_Os07g17390.1				✔				-1.34
LOC_Os07g19444.1				✔			-2.29	
LOC_Os07g25450.1	✔						-1.10	1.47
LOC_Os07g32380.1			✔				-1.16	
LOC_Os07g32420.1			✔					1.09
LOC_Os07g32420.2			✔					1.09
LOC_Os07g32420.3			✔					1.09
LOC_Os07g32430.1		✔						1.66
LOC_Os07g33320.1						✔	-3.91	
LOC_Os07g35260.1					✔			-1.53
LOC_Os07g35260.2					✔			-1.53
LOC_Os07g35310.1					✔			2.30
LOC_Os07g35310.2					✔			2.30
LOC_Os07g35680.1		✔						-2.38
LOC_Os07g36170.1						✔		1.64
LOC_Os07g36170.2						✔		1.64
LOC_Os07g37610.1	✔							1.55
LOC_Os07g39270.1	✔						1.30	
LOC_Os07g39270.2	✔						1.30	
LOC_Os07g41720.1	✔						-2.70	
LOC_Os07g41720.2	✔						-2.70	
LOC_Os07g42420.1			✔				-10.12	-2.55
LOC_Os07g42520.1	✔						-1.36	1.52
LOC_Os07g42910.1						✔		3.24
LOC_Os07g43604.1		✔						1.32

LOC_Os07g43730.1				✔			-1.30	
LOC_Os07g44410.1	✔						3.83	2.52
LOC_Os07g44440.1	✔						4.23	
LOC_Os07g44480.1					✔		-8.87	
LOC_Os07g48870.1	✔						1.99	
LOC_Os08g02180.1		✔						1.92
LOC_Os08g03310.1	✔							-1.46
LOC_Os08g03310.2	✔							-1.46
LOC_Os08g03310.3	✔							-1.46
LOC_Os08g03310.4	✔							-1.46
LOC_Os08g03310.5	✔							-1.46
LOC_Os08g03310.6	✔							-1.46
LOC_Os08g06380.1						✔	-2.54	
LOC_Os08g06640.1	✔							1.95
LOC_Os08g09610.1					✔			-1.63
LOC_Os08g10300.1			✔				-1.71	
LOC_Os08g10310.1		✔					-3.60	-1.22
LOC_Os08g10310.2		✔					-3.60	-1.22
LOC_Os08g10310.3		✔					-3.60	-1.22
LOC_Os08g13699.1		✔					-1.43	
LOC_Os08g13890.1			✔					1.16
LOC_Os08g14620.1			✔				-1.58	1.00
LOC_Os08g16350.1	✔						-1.85	
LOC_Os08g16350.2	✔						-1.85	
LOC_Os08g16350.3	✔						-1.85	
LOC_Os08g18044.1				✔			-1.54	
LOC_Os08g18044.2				✔			-1.54	
LOC_Os08g19370.1			✔					1.40
LOC_Os08g19374.1		✔					1.65	
LOC_Os08g19374.2		✔					1.65	
LOC_Os08g19374.3		✔					1.65	
LOC_Os08g19374.4		✔					1.65	
LOC_Os08g19670.1				✔				-1.02
LOC_Os08g19830.1				✔			-1.84	
LOC_Os08g19830.3				✔			-1.84	
LOC_Os08g23730.1				✔			-1.77	
LOC_Os08g25390.1			✔					-1.38
LOC_Os08g25390.2			✔					-1.38
LOC_Os08g25590.1	✔						-1.23	
LOC_Os08g25590.2	✔						-1.23	
LOC_Os08g25590.3	✔						-1.23	
LOC_Os08g26880.1		✔						-3.15
LOC_Os08g29170.1			✔					-1.18
LOC_Os08g29590.1				✔			1.03	-1.04
LOC_Os08g30634.1			✔				-7.71	

LOC_Os08g30634.2			✔				-7.71	
LOC_Os08g30634.3			✔				-7.71	
LOC_Os08g31140.1					✔			3.67
LOC_Os08g31814.1	✔							1.54
LOC_Os08g31814.2	✔							1.54
LOC_Os08g33400.1				✔				-1.13
LOC_Os08g33400.2				✔				-1.13
LOC_Os08g34060.1				✔			-1.72	1.35
LOC_Os08g34060.2				✔			-1.72	1.35
LOC_Os08g34060.3				✔			-1.72	1.35
LOC_Os08g35870.1	✔						-1.61	
LOC_Os08g35870.2	✔						-1.61	
LOC_Os08g43480.1	✔						-1.10	2.16
LOC_Os08g43480.2	✔						-1.10	2.16
LOC_Os09g02214.1	✔						-3.14	
LOC_Os09g02214.2	✔						-3.14	
LOC_Os09g02214.3	✔						-3.14	
LOC_Os09g02270.1					✔		-1.08	
LOC_Os09g02270.2					✔		-1.08	
LOC_Os09g04790.1	✔						1.35	1.80
LOC_Os09g06740.1			✔				-1.35	
LOC_Os09g10054.1				✔			-1.71	
LOC_Os09g13440.1		✔					8.74	3.16
LOC_Os09g13440.2	✔						8.74	3.16
LOC_Os09g13890.1		✔					-1.47	
LOC_Os09g16920.1		✔					-2.50	
LOC_Os09g19229.1		✔						-1.16
LOC_Os09g20490.1	✔						-2.17	
LOC_Os09g25550.1			✔					-1.63
LOC_Os09g25880.1		✔				✔	-2.69	
LOC_Os09g27795.1				✔			-1.63	
LOC_Os09g29284.1	✔						-8.51	
LOC_Os09g31080.1	✔						-2.42	
LOC_Os09g31080.3	✔						-2.42	
LOC_Os09g31080.4	✔						-2.42	
LOC_Os09g31478.1			✔				2.27	2.19
LOC_Os09g31478.2			✔				2.27	2.19
LOC_Os09g31482.1	✔							1.77
LOC_Os09g31482.3	✔							1.77
LOC_Os09g31502.1		✔						-1.83
LOC_Os09g31502.2		✔						-1.83
LOC_Os09g31502.4		✔						-1.83
LOC_Os09g32169.1			✔					1.30
LOC_Os09g32200.1		✔						-2.47
LOC_Os09g34150.1				✔			3.13	

LOC_Os09g34150.2				✔			3.13	
LOC_Os09g34320.1						✔	-2.81	
LOC_Os09g36250.1			✔				-2.35	
LOC_Os10g01610.1	✔							1.02
LOC_Os10g02240.1						✔		-1.80
LOC_Os10g02350.1		✔					-1.18	
LOC_Os10g02490.1		✔					-8.37	-1.92
LOC_Os10g02490.2		✔					-8.37	-1.92
LOC_Os10g02500.1	✔						-1.91	
LOC_Os10g04342.1	✔						-3.25	
LOC_Os10g05690.1		✔					-2.46	
LOC_Os10g11310.1		✔						-1.82
LOC_Os10g11310.2		✔						-1.82
LOC_Os10g11310.3		✔						-1.82
LOC_Os10g11810.1			✔					1.10
LOC_Os10g11810.2			✔					1.10
LOC_Os10g11810.3			✔					1.10
LOC_Os10g11889.1				✔			-4.53	
LOC_Os10g11889.2				✔			-4.53	
LOC_Os10g12500.1		✔					-2.00	
LOC_Os10g16640.1		✔					-2.22	
LOC_Os10g16640.2		✔					-2.22	
LOC_Os10g17660.1				✔			-8.44	
LOC_Os10g20260.1	✔						-2.67	
LOC_Os10g22460.1		✔					-1.37	
LOC_Os10g22520.1		✔					2.34	
LOC_Os10g22570.1					✔		-2.43	
LOC_Os10g25674.1		✔						1.30
LOC_Os10g25674.2		✔						1.30
LOC_Os10g27090.1						✔	-3.14	
LOC_Os10g27274.1		✔					-2.76	
LOC_Os10g27274.2		✔					-2.76	
LOC_Os10g27274.3		✔					-2.76	
LOC_Os10g31500.1	✔						-8.70	
LOC_Os10g32680.1				✔			1.15	2.34
LOC_Os10g32680.2				✔			1.15	2.34
LOC_Os10g32680.3				✔			1.15	2.34
LOC_Os10g32700.1					✔		1.73	
LOC_Os10g33140.1						✔		1.55
LOC_Os10g35110.1						✔		1.80
LOC_Os10g35460.1			✔					1.87
LOC_Os10g37730.1						✔	-1.09	
LOC_Os10g37730.2						✔	-1.09	
LOC_Os10g37730.3						✔	-1.09	
LOC_Os10g38450.1			✔				3.33	

LOC_Os10g38470.1	✔						2.28	2.09
LOC_Os10g38870.1			✔				-1.12	1.61
LOC_Os10g38890.1		✔						1.24
LOC_Os10g39090.1	✔						-1.14	
LOC_Os10g39090.2	✔						-1.14	
LOC_Os10g40614.1	✔						-2.36	
LOC_Os10g41310.1			✔				-1.82	
LOC_Os10g41390.1		✔					-8.12	
LOC_Os10g41560.1		✔					-2.31	2.09
LOC_Os10g42439.1			✔					-1.58
LOC_Os11g02020.1				✔			-7.71	
LOC_Os11g04370.1		✔					-3.31	
LOC_Os11g04520.2		✔						1.90
LOC_Os11g06690.1		✔						-1.59
LOC_Os11g08100.1			✔				-1.20	1.33
LOC_Os11g08110.1		✔					-3.64	
LOC_Os11g08110.2		✔					-3.64	
LOC_Os11g08380.1		✔					-3.29	
LOC_Os11g08940.1				✔			-2.14	
LOC_Os11g10090.1					✔		-2.16	
LOC_Os11g10310.1					✔			-1.73
LOC_Os11g20040.1	✔						-4.40	
LOC_Os11g20170.1						✔	-1.56	
LOC_Os11g24060.1						✔	-2.47	
LOC_Os11g24140.1		✔					2.58	
LOC_Os11g29120.1		✔					-1.27	
LOC_Os11g29120.2		✔					-1.27	
LOC_Os11g29400.1	✔						-1.29	
LOC_Os11g29900.1						✔	-1.41	1.08
LOC_Os11g29900.2						✔	-1.41	1.08
LOC_Os11g32320.1					✔			1.95
LOC_Os11g33120.1		✔						1.04
LOC_Os11g33120.2		✔						1.04
LOC_Os11g33120.3		✔						1.04
LOC_Os11g33394.1	✔						-2.70	
LOC_Os11g33394.2	✔						-2.70	
LOC_Os11g35920.1					✔		-3.23	
LOC_Os11g36719.1		✔					-8.03	
LOC_Os11g37860.1						✔	1.56	
LOC_Os11g37960.1	✔						2.29	
LOC_Os11g38260.1		✔						-1.07
LOC_Os11g38900.1				✔				1.01
LOC_Os11g41160.1			✔					-1.61
LOC_Os11g41160.2			✔					-1.61
LOC_Os11g41160.3			✔					-1.61

LOC_Os11g41600.1						✔	2.07	1.63
LOC_Os11g41600.2						✔	2.07	1.63
LOC_Os11g42000.1					✔		-3.21	
LOC_Os11g43895.1			✔				-2.45	
LOC_Os11g44810.1			✔					1.73
LOC_Os11g44810.2			✔					1.73
LOC_Os11g44810.3			✔					1.73
LOC_Os12g08260.1				✔			2.62	2.44
LOC_Os12g08260.2				✔			2.62	2.44
LOC_Os12g08260.3				✔			2.62	2.44
LOC_Os12g08260.4				✔			2.62	2.44
LOC_Os12g08260.5				✔			2.62	2.44
LOC_Os12g08260.6				✔			2.62	2.44
LOC_Os12g08260.7				✔			2.62	2.44
LOC_Os12g09550.1		✔					-4.29	
LOC_Os12g09550.2		✔					-4.29	
LOC_Os12g09580.1			✔					-2.35
LOC_Os12g16010.1				✔			-1.06	
LOC_Os12g16080.1			✔		✔		2.67	3.81
LOC_Os12g16410.1			✔				-8.37	
LOC_Os12g16410.2			✔				-8.37	
LOC_Os12g16410.3			✔				-8.37	
LOC_Os12g16690.1				✔			-9.79	
LOC_Os12g17950.1				✔			-1.06	
LOC_Os12g18360.1			✔					3.43
LOC_Os12g18360.2			✔					3.43
LOC_Os12g19370.1			✔				-4.32	
LOC_Os12g19381.1	✔							-1.15
LOC_Os12g20144.1			✔				-2.17	
LOC_Os12g24020.1				✔			-1.47	4.26
LOC_Os12g24020.2				✔			-1.47	4.26
LOC_Os12g24020.3				✔			-1.47	4.26
LOC_Os12g24170.1	✔						-1.09	1.03
LOC_Os12g24250.1			✔				-2.02	
LOC_Os12g25690.1			✔				-2.76	
LOC_Os12g27830.1			✔				8.54	
LOC_Os12g28015.1						✔		1.22
LOC_Os12g28770.1						✔	-2.23	
LOC_Os12g30180.1					✔		2.39	
LOC_Os12g34330.1	✔						-1.70	
LOC_Os12g34500.1	✔						-3.28	
LOC_Os12g36930.1				✔				-1.03
LOC_Os12g37910.1	✔							-1.11
LOC_Os12g37910.2	✔							-1.11
LOC_Os12g38140.1						✔	-3.52	

LOC_Os12g39980.1		✔					-9.95	
LOC_Os12g41180.1			✔				-3.16	
LOC_Os12g41710.1		✔			✔			2.31
LOC_Os12g41710.2		✔			✔			2.31
LOC_Os12g41710.3		✔			✔			2.31
LOC_Os12g41710.4		✔			✔			2.31
LOC_Os12g42730.1				✔				2.22
LOC_Os12g42730.2				✔				2.22
LOC_Os12g42730.3					✔			2.22
LOC_Os12g42730.4					✔			2.22

Supplementary Table S6. Summary of all MS^2 data for metabolites of atrazine in rice

No.	Acronym	Chemical formula	t_R[a]	Theor *m/z* $[M+H]^+$	Exptl *m/z* $[M+H]^+$	Delta (ppm)	MS^2 main fragments[b] (*m/z*)
#-1	AME	$C_9H_{17}N_5S$	8.19	228.1277	228.1286	-3.95	**186**, 141, 96
#-2	ATR	$C_8H_{14}ClN_5$	11.46	216.101	216.1011	-0.46	**174**, 145, 132
Metabolites of atrazine							
1	HIA	$C_8H_{14}ClN_5O$	4.26	232.096	232.0955	2.15	**214**, 174, 136, 120, 107
2	DHA	$C_8H_{12}ClN_5$	5.74	214.0854	214.0849	2.34	**172**, 130, 105
3	Atraton	$C_9H_{17}N_5O$	4.31	212.1506	212.1499	3.30	**170**, 167
4	DMA	$C_7H_{12}ClN_5$	7.53	202.0854	202.0849	2.47	174, 132, **124**
5	HA	$C_8H_{15}N_5O$	2.07	198.1349	198.1342	3.53	**156**, 114
6	DEA	$C_6H_{10}ClN_5$	3.71	188.0697	188.0701	-2.13	174, **146**, 110, 104
7	DIA	$C_5H_8ClN_5$	2.43	174.0541	174.0538	1.72	146, 132, **118**, 104
8	DACT	$C_6H_{10}ClN_5$	1.66	146.0228	146.0229	-0.68	128, 121, **114**
Conjugates of atrazine							
1	DHA+Cys & Ser+GlcN-H_2O[c]	$C_{20}H_{35}ClN_8O_9S$	8.42	599.2009	599.2006	0.50	469, **333**, 299, 267, 249
2	ATR-HCl+GSH	$C_{18}H_{30}N_8O_6S$	2.82	487.2082	487.2061	4.31	358, **214**, 172, 145
3	ATR-HCl+(Cys & Glu)	$C_{16}H_{27}N_7O_5S$	2.78	430.1867	430.1852	3.49	301, **214**, 172
4	ATR-HCl+(Cys & Ser)[c]	$C_{14}H_{25}N_7O_4S$	1.76	388.1761	388.1751	2.58	299, **267**, 223
5	ATR-HCl+(Cys & Gly)	$C_{13}H_{23}N_7O_3S$	2.83	358.1656	358.1642	3.91	301, **214**
6	DHA+Cys	$C_{11}H_{19}ClN_6O_2S$	6.48	335.1051	335.1056	-1.49	317, **214**
7	DIHA+Glc-H_2O	$C_{11}H_{19}N_5O_6$	2.01	318.1408	318.1411	-0.94	**156**
8	ATR-HCl+CH_3+Cys	$C_{12}H_{22}N_6O_2S$	5.33	315.1598	315.1596	0.63	**267**, 197, 151
9	ATR-HCl+Cys	$C_{11}H_{20}N_6O_2S$	3.08	301.1441	301.1426	4.98	284, **214**, 172, 130

#-1, internal standard; #-2, chemical technical; t_R[a], retention time; MS^2 main fragments[b], base peak of MS^2 fragment ions are shown in bold. [c] Compounds that have been reported for the first time.

Supplementary Table S7. Primer sequences used for this study

	Primer name	Forward (5'-3')	Reverse (5'-3')
qRT-PCR	LOC_Os06g13560.1	CCAGGCTCCTACTACACCA	GATCATCTGAGAACGCCAAT
	LOC_Os06g37300.1	AAGAGTTTGGGAGGGAGATA	TTCCAGCAAGAAGTCAATGTAG
	LOC_Os10g38470.1	GCCTTCAACGACGACACGC	CCCTTGGAGCACTCCCTCA
	LOC_Os03g28940.1	TTGATGACTTCCCAGCTGAGAA	GCGCTGTGGAGGAACTCTTG
	LOC_Os07g48870.1	GGTGGCTGAACTACCTGAG	TTGATCTCGTTGTCGGTGC
	LOC_Os05g41080.1	AACATCGGCTTCAGGGACG	GATCAGCAGTTCGGTGGTT
	LOC_Os12g18360.1	ATCGACTCCTTGAAACTATCC	AGTATTCTACTGCAACTATTATTATC
	LOC_Os02g12380.1	ACAAACCTATGGGACACTCA	CCATCGGTTCTGGTACTTTA
	LOC_Os02g19924.1	CATTTGTTCAGGGAGCATT	CTCTATGCCATCTAGGGAAG
	LOC_Os05g34854.2	CCGATTACTTCTCCACCCTA	GTCCTCGAAGAACTCCCTGT
	LOC_Os03g40540.1	TCAAGCAGGGACCCAGTTTC	TCAGCTCCGCCTCCATACAC
	OsRDR1	TGCTTCTCGCCAGGGATTAG	TCCACCTCATACTTCTGAACCT
	LOC_Os03g02010	AAGTTCGAGTGGGACACAGACG	TTGGCCTTCCCATTTGCATCCTG
	LOC_Os05g37350	CGACGGCGTTGCTGAATACC	GCAGGAAGGTTGTAGGATGGC
	LOC_Os10g42690	CTACCAAGGAGGAGTTCGAGGAT	TCAACACGACACCAGCAGGAA
	LOC_Os03g58400	TAGAGGCAGGGGCTTATGGT	TTGACAGCGGCGTAGAACTT
	LOC_Os01g70220	ACCTGCTGCCGAGGATGATGT	GCCTTCCTGTAACACCGCTTGT
	LOC_Os09g13740	GGATGTTGTTCTCTTGTTGTTCGTC	CCCATTGCCACATTTCTCTGTCTTA
	OsActin	GAGTATGATGAGTCGGGTCCAG	ACACCAACAATCCCAAACAGAG
RT-PCR	OsRDR1	AGCGACATTAGGACATGGATGG	TGGCTGGTACTTGCTGTATGC
	LOC_Os03g02010	AGAGGTTAAGGTCAGTCCACAGA	TTCCTACATTCGTCCAAGACATACT
	LOC_Os05g37350	CGACGGCGTTGCTGAATACC	ATGGACCTCGAAGCGACAAGAA
	LOC_Os10g42690	CCTGCTGGTGTCGTGTTGATG	TCTGTTACTCCTGGAATTGGTGTCT
	LOC_Os03g58400	GCAAGTTCTACGCCGCTGTCAAGA	GGATGGAAGCACATGCCGACCTT
	LOC_Os01g70220	CACCAGCCATTACCTTCACCTT	TTCCACGTTGCAGTTGTTGAGA
	LOC_Os09g13740	AGAAATGGACTGTTGAGGGAGAGA	ATATACTTGCTGCGAGATACATGCT
	LOC_Os09g02214.2	TGATGTGACGACCACTATGTTGT	TCCTTGTGTATCAATCCGTGACA
	LOC_Os06g37300.1	AAAAGTATGGTCGCAATCAG	GGAAGTAGTCCCTCCAGTCA
	LOC_Os10g38470.1	GCCTTCAACGACGACACGC	TGCAGCACCTTGCCGAACT
	LOC_Os03g28940.1	AGCCGATGCGAACAAGG	CAGCAGCATTTGGACGAAC
	LOC_Os07g48870.1	CGGCTGCGGTGGCTGAACTA	GCGACGCTCATCTGTGGTGC
	OsUbiquitin	CGCAAGTACAACCAGGACAA	TGGTTGCTGTGACCACACTT
MSP-PCR	LOC_Os10g38470.1	GAAAATTTGTAGTTGATGGGAT	CATCTTCTTCAACAACTAAAAAA

• Supplementary Note

Description of effect of DNA methylation inhibitors on accumulation of ATR residues and its metabolites (conjugates) in rice

Under positive ionization, the standard ATR chromatogram showed a retention time of 11.46 min (Fig. S11A), which was confirmed by the presence of $[M+H]^+$ ions with *m/z* 216 (Fig. S12B). Decomposition of *m/z* 216 precursor ions upon MS/MS analysis produced three fragment ions at *m/z* 174, *m/z* 145 and *m/z* 132 (Fig. S12B). Hydroxyisopropyl atrazine (HIA, metabolite #1), which peaked at 3.89 min was confirmed by loss of H_2O and isopropenyl to form *m/z* 214 and *m/z* 174, and the loss of chlorine to form *m/z* 136 from precursor ion of *m/z* 174 (Fig. S11B and S12C). For metabolite #2 (*m/z* 214), which peaked at 5.74 min, the fragment ion of *m/z* 172 from the loss of isopropenylacetylene indicated that the double bond occurred on the ethyl group (Fig. S11B and S12D). The fragment ion of *m/z* 130 was generated by the loss of $-N-CH=CH_2$ from *m/z* 172. Metabolite #2 was identified as dehydrogenated atrazine (DHA). The mass spectrum of metabolite #3 peaked at 4.31 min, and the fragment ion of m/z 212 was produced by loss of chlorine and addition of methoxy group from ATR (Fig. S11C and S12E). Its main product ions were m/z 170 and m/z 158, which was the loss of isopropyl and $-NH-CH_2-CH_3$ groups, respectively. Thus, metabolite #3 was identified as a triazine herbicide (Atraton), which was more toxic than atrazine[1]. Demethylated atrazine (DMA, metabolite #4), with the elimination of $-CH_3$ (14 Da) and further elimination of $-NH_2-CH_2-CH_3$ (42 Da), was identified according to the fragment ion of *m/z* 174 and m/z 132 (Fig. S11C and S12F). Hydroxyatrazine (HA, metabolite #5), which peaked at 2.07 min, was derived from atrazine by substitution of a hydroxyl group for the chlorine, while the loss of isopropenylacetylene and $-NH-CH_2-CH_3$ groups resulted in formation of fragment ions of *m/z* 156 and *m/z* 114, respectively (Fig. S11D and S12G). Metabolite #6 peaked at 3.71 min with two

main fragment ions at *m/z* 174/146 which were generated by the cleavage of $-CH_3$ and isopropyl groups (Fig. S11D and S12H). Base on the mass spectra, metabolite #6 was desethylated atrazine (DEA). From the accurate mass value of $[M+H]^+$ ion (*m/z* 174) and its fragments at *m/z* 146/132/118/104, it can be determined that the metabolite #7 was deisopropylated atrazine (DIA), *N*-demethylated from DMA (Fig. S11E and S12I). Beside the products, a major metabolite was detected with a retention time of 1.66 min (Fig. S11E). The information of MS/MS spectra showed that the elimination of amino group led to occurrence of fragment ions at *m/z* 128, from which metabolite #8 as didealkyl atrazine (DACT) (Fig. S12J).

Compared to the controls (ATR-free) and treatments (ATR- and AZA+ATR-), seven atrazine-glutathione metabolites and two glucosylated-atrazine conjugates were identified. The peak at 8.42 min was detected and revealed a glucosylated ATR-derivative (Conjugate #1) with main $[M+H]^+$ ions at *m/z* 333/299/267/249 (Fig. S11F and S13A). Ion of *m/z* 333 was attributed to the elimination of Cys-*β*-Ser (175 Da) and $Y^{1,4}$ ion (89 Da) groups from its precursor. Subsequently, the loss of chlorine, thiol and hydroxyl groups from *m/z* 333 led to the formation of *m/z* 299, *m/z* 267 and *m/z* 249, respectively, suggesting that the conjugate was identified as cysteinyl β-serine and glucosamine conjugate by dehydrogenated atrazine (DHA+Cys & Ser+GlcN-H_2O, conjugate #1). A protonated molecule (*m/z* 487, t_R=2.82 min) was created by conjugation (Fig. S11F and S13B). The loss of glutamic acid (Glu) for *m/z* 487 formed the fragment ion of *m/z* 358, and the cleavage of thiol group generated the fragment ion of *m/z* 214. These data were consistent with the conjugation of ATR and glutathione (GSH) (ATR-HCl+GSH, conjugate #2). Conjugate #3 (*m/z* 430, t_R=2.78 min) was determined by its fragment ions at m/z 301/214/172, suggesting that the compound was created from conjugate #2 by the hydrolysis of peptide linkage between glycine and cysteine (Fig. S11G and S13C). Thus, conjugate #3 was identified as cysteinyl γ-glutamic acid conjugate by

chlorine substitution for atrazine (ATR-HCl+Cys & Glu). A major ATR conjugate (*m/z* 388) eluting at 1.76 min was detected according to MS^2 (Fig. S11G). The fragment ion of *m/z* 299 was the result of the loss of serine. Subsequently the loss of imine and hydroxy groups from precursor ion (*m/z* 299) generated the fragment ion of *m/z* 267, confirming that of from conjugate #3, conjugate #5 (ATR) was cysteinyl *β*-serine conjugate by chlorine substitution for atrazine (ATR-HCl+Cys & Ser) (Fig. S13D). The conjugate #5 (*m/z* 358) peaking at 2.83 min were detected on the basis of the fragment ions of *m/z* 301/214, suggesting that cysteinyl *β*-glycine linked to ATR by thiol bond (Fig. S11H and S13E). So the parent ion of *m/z* 358 should point to ATR-HCl+(Cys & Gly). As shown in Fig. S13F, the MS^2 data with fragment ion of *m/z* 214 indicated that the compound was formed by conjugation of DHA and cysteine. The loss of H_2O from the parent ion of *m/z* 335 formed the fragment ion of *m/z* 317. Thus, the conjugates #6 at 6.48 min was confirmed as a cysteine conjugate for DHA (DHA+Cys) (Fig. S11H). The peak at 2.01 min was detected by MS and revealed a glucosylated ATR-derivative (conjugate #7) with $[M+H]^+$ ions at *m/z* 318 (Fig. S11H and S13F). The precursor ion yielded characteristic fragment ion at *m/z* 156, which could be attributed to the loss of a glucose moiety (162 Da). Thus, the parent ion of *m/z* 318 was assigned to DIHA+Glc-H_2O, a sugar ring conjugate by substation for dimethyl hydroxyl atrazine via the *O*-glycosidic bond. A peak (t_R =5.33 min) at *m/z* 315 was detected (Fig. S11I and S13G). The MS^2 spectrum generated from *m/z* 315 ion showed fragment ions at *m/z* 267, *m/z* 197 and *m/z* 151. Elimination of hydroxyl, amino and methyl groups from *m/z* 315 resulted in the formation of *m/z* 267 ion. Cleavage of −CH_2−S− bond and loss of isopropyl group led to the formation of *m/z* 197 ion, suggesting that a cysteine linked to methylated atrazine. The *m/z* 151 was created by the loss of thiol group from the precursor ion (*m/z* 197) (Fig. S13G). A major ATR conjugate (*m/z* 301) eluting at 4.98 min with stronger intensity than other conjugates was detected according to MS^2 (Fig. S11I, S13H). For *m/z*

301, the loss of NH_3 produced the fragment ion of m/z 284. The MS^2 data with the fragment ions of m/z 214 and m/z 172 showed that a protonated thiol atrazine generated (Fig. S13H). The conjugate was clearly identified as a cysteine conjugate by chlorine substitution for atrazine (ATR-HCl+Cys, conjugate #9).

Our recent study characterized several atrazine metabolites in alfalfa (*Medicago sativa* L.)[2], including that hydroxyisopropyl atrazine (HIA, metabolite #1), dehydrogenated atrazine (DHA, metabolite #2), demethylated atrazine (DMA, metabolite #4), hydroxyatrazine (HA, metabolite #5), desethylated atrazine (DEA, metabolite #6), DHA+Cys (conjugate #6), ATR-HCl+CH_3+Cys (conjugate #8) and ATR-HCl+Cys (conjugate #9). Beside these metabolites, we found metabolite #3 (Atraton), metabolite #8 (DACT), conjugate #2 (ATR-HCl+GSH), conjugate #3 (ATR-HCl+Cys & Glu), conjugate #5 (ATR-HCl+Cys & Gly) and conjugate #7 (DIHA+Glc-H_2O) was reported in rice for the first time. Moreover, conjugate #1 and conjugate #4, were reported in plant for the first time.

References

1. Tchounwou, P. B. *et al*. Toxicity assessment of atrazine and related triazine compounds in the microtox assay, and computational modeling for their structure-activity relationship. *Int. J. Mol. Sci.* **1**: 63−74 (2000).
2. Zhang J. J, Lu Y. C. & Yang H. Chemical modification and degradation of Atrazine in Medicago sativa through multiple pathways. *J. Agr. Food Chem.* **62**: 9657−9668 (2014).
